# The β3‐AR agonist BRL37344 ameliorates the main symptoms of X‐linked nephrogenic diabetes insipidus in the mouse model of the disease

**DOI:** 10.1111/jcmm.18301

**Published:** 2024-04-23

**Authors:** Serena Milano, Ilenia Saponara, Andrea Gerbino, Monica Carmosino, Maria Svelto, Giuseppe Procino

**Affiliations:** ^1^ Department of Biosciences, Biotechnologies and Environment University of Bari Bari Italy; ^2^ Department of Sciences University of Basilicata Potenza Italy

**Keywords:** (3–6) vasopressin, antidiuresis, beta‐3 adrenoreceptor, BRL37344, kidney, nephrogenic diabetes insipidus

## Abstract

X‐linked nephrogenic diabetes insipidus (X‐NDI) is a rare congenital disease caused by inactivating mutations of the vasopressin type‐2 receptor (AVPR2), characterized by impaired renal concentrating ability, dramatic polyuria, polydipsia and risk of dehydration. The disease, which still lacks a cure, could benefit from the pharmacologic stimulation of other GPCRs, activating the cAMP‐intracellular pathway in the kidney cells expressing the AVPR2. On the basis of our previous studies, we here hypothesized that the β3‐adrenergic receptor could be such an ideal candidate. We evaluated the effect of continuous 24 h stimulation of the β3‐AR with the agonist BRL37344 and assessed the effects on urine output, urine osmolarity, water intake and the abundance and activation of the key renal water and electrolyte transporters, in the mouse model of X‐NDI. Here we demonstrate that the β3‐AR agonism exhibits a potent antidiuretic effect. The strong improvement in symptoms of X‐NDI produced by a single i.p. injection of BRL37344 (1 mg/kg) was limited to 3 h but repeated administrations in the 24 h, mimicking the effect of a slow‐release preparation, promoted a sustained antidiuretic effect, reducing the 24 h urine output by 27%, increasing urine osmolarity by 25% and reducing the water intake by 20%. At the molecular level, we show that BRL37344 acted by increasing the phosphorylation of NKCC2, NCC and AQP2 in the renal cell membrane, thereby increasing electrolytes and water reabsorption in the kidney tubule of X‐NDI mice. Taken together, these data suggest that human β3‐AR agonists might represent an effective possible treatment strategy for X‐NDI.

## 1 INTRODUCTION

In the kidney, the binding of arginine‐vasopressin (AVP) to its type‐2 receptor (AVPR2), via cAMP signalling, triggers antidiuresis by increasing the apical plasma membrane expression of the water channel AQP2 in the collecting duct (CD),^1^ the apical translocation and phosphorylation of the NKCC2 co‐transporter in the thick ascending limb (TAL),^2^ and the phosphorylation/activation of the NCC co‐transporter in the distal convolute tubule (DCT).^3^ Reabsorption of NaCl through NKCC2 and NCC generates and maintains the cortical‐medullary osmotic gradient providing the driving force for the AVP‐dependent reabsorption of water into CD through AQP2.

X‐linked nephrogenic diabetes insipidus (X‐NDI) is a rare genetic disorder caused by loss‐of‐function mutations in the gene encoding AVPR2.^4^ Estimates of the incidence of congenital NDI indicate 8.8 per million male live births.^5^ Ninety percent of all forms of NDI are X‐linked, while approximately the remaining 10% are due to loss‐of‐function mutations in the AQP2 gene.^6^, ^7^, ^8^, ^9^, ^10^ Given the substantial expression of the AVPR2 receptor in the TAL, DCT, CNT and CD,^11^, ^12^, ^13^ involved in the reabsorption of water and solutes from the glomerular filtrate, the renal phenotype in X‐NDI is characterized by severe polyuria (often >15 L/day), hyposthenuria, compensatory polydipsia, hypernatremia and increased plasma osmolarity. The disease has a severe impact on the quality of life due to the continuous urge to empty the bladder, even during sleep, the equally frequent need to drink water and the need to plan daily activities according to these needs.

Currently, X‐NDI‐related symptoms are managed with continuous water supply, a low‐sodium and low‐protein diet to reduce osmotic load,^5^ and a combination of drugs, such as thiazide, amiloride and prostaglandin synthesis inhibitors.^14^, ^15^ The effects of the above treatment on the reduction of polyuria are, however, modest and the side effects related to the drugs used are not negligible.

Examining the spectrum of possible novel pharmacological approaches tested in preclinical studies (see Mortensen et al.^16^ for review), stimulation of other GPCRs, triggering the cAMP‐mediated signalling pathway in renal cells with defective AVPR2, has emerged as a promising strategy for finding a cure for X‐NDI.

A few years ago, we reported that the beta‐3 adrenergic receptor (β3‐AR) is co‐expressed with the AVPR2 in the mouse kidney tubule cells of the TAL, DCT, CNT and cortical and outer medullary CD and activates the cAMP/PKA signalling pathway.^17^ We also showed ex vivo that in mouse kidney slices, the specific agonism of β3‐AR with BRL37344 induces AQP2 apical accumulation in CD principal cells, NKCC2 phosphorylation in the TAL,^17^ and NCC phosphorylation in the apical membrane of DCT cells.^18^ In vivo, we found that β3‐AR^−/−^ mice show mild polyuria and reduced urine osmolarity compared to wt mice, paralleled by reduced plasma membrane expression of AQP2 and reduced phosphorylation of NKCC2^17^ and NCC.^18^ In wt, but not in β3‐AR^−/−^ mice, a single i.p. injection of the β3‐AR agonist BRL37344 0.6 mg/kg triggered a short‐term antidiuretic effect and, more importantly, the same effect was observed in the mouse model of X‐NDI.^17^ Therefore, in the present work, we studied the effect of chronic stimulation of β3‐AR in the mouse model of X‐NDI, showing that continuous administration of BRL37344 dramatically corrected the polyuria, the hyposthenuria and the compensatory polydipsia of these animals throughout the 24 h, likely by promoting tubular reabsorption of NaCl, through the activation of NKCC2 and NCC, and water, through AQP2. Overall, these findings suggest that β3‐AR agonists have the potential to serve as an effective therapeutic strategy for treating X‐NDI by compensating for the defective AVPR2 function and restoring water and solute reabsorption in the renal tubules.

## 2 MATERIALS AND METHODS

### 2.1 Antibodies and reagents

Antibodies anti‐NKCC2 were from Merck Millipore (AB3562P, Merck Millipore, Burlington, MA). The antibody against the phosphorylated threonine 96 and 101 NKCC2^19^ was kindly provided by Prof. Biff Forbush, Yale University. Antibodies anti‐NCC were from StressMarq Biosciences Inc. (SPC‐402D, StressMarq Biosciences Inc., Victoria, BC, Canada). Antibodies against the phosphorylated Thr 53 NCC were from Phosphosolutions (p1311‐53, Phosphosolutions, Aurora, CO). The antibody against human AQP2 was previously described.^20^ Antibodies against the phosphorylated 256 AQP2 were from Abcam (ab111346, Abcam, Cambridge, UK). BRL37344 was from Santa Cruz Biotechnology (sc‐200154, Santa Cruz Biotechnology, Dallas, TX).

#### 2.1.1 Animal studies

Procedures involving animals were carried out in compliance with the Italian guidelines for animal care (DL 26/14) and the European Communities Council Directive (2010/63/UE). Procedures were approved by the Ethical Committee in Animal Experiments of the University of Bari (study # F2AEC.9.EXT.4.).

The conditional mouse model of X‐linked Nephrogenic Diabetes Insipidus has been described previously.^21^ Mice were maintained on a 12‐h light/12‐h dark cycle, with free access to water and food, in accordance with the Italian Institute of Health Guide for the Care and Use of Laboratory Animals. Mice were fed with a standard mouse chow (Tekland 2018, Envigo, Indianapolis, IN). All studies were carried out with male maintained on a C57BL/6J background and the induction of type2‐vasopressin receptor gene deletion was performed as previously reported.^22^ Briefly, 7‐week‐old male AVPR2^fl/y^ Esr1‐Cre were given a daily intra‐peritoneal injection (i.p.) of 4‐OH‐TMX (0.1 mL of a 5 mg/mL suspension) for 6 days. Animals were treated with BRL37344 5 months after the last TMX injection. All mice (AVPR2^fl/y^ Esr1‐Cre) which showed, after AVPR2 genetic ablation induced by tamoxifen administration, urine output ≥10 mL/day and urine osmolality ≤700 mOsm/kg were included in the study.

For each experimental protocol, two different investigators were involved: a first investigator (IS) loaded syringes with the vehicle or BRL37344, collected urine samples and performed measurements of urine volume and osmolality. A second investigator (SM), unaware of treatment, injected mice and performed data analysis. The primary outcome of the study was a significant reduction of the urine output. To study the effects of BRL37344 administration on urinary parameters, mice were single‐housed in metabolic cages to collect urine and measure water and food consumption.

#### 2.1.2 Treatment with BRL37344

All X‐NDI mice used in experiments were male and age‐matched. All parameters (urine volume and osmolarity, urine electrolytes and creatinine, water and food consumption) were recorded for 5 days before the injections and were used to calculate the baseline for each mouse. 16 X‐NDI were randomized in two groups: CTR group (*n* = 5) received i.p. of saline (vehicle), BRL group (*n* = 11) received i.p. of BRL37334.

#### 2.1.3 Protocol 1

**FIGURE 1:**
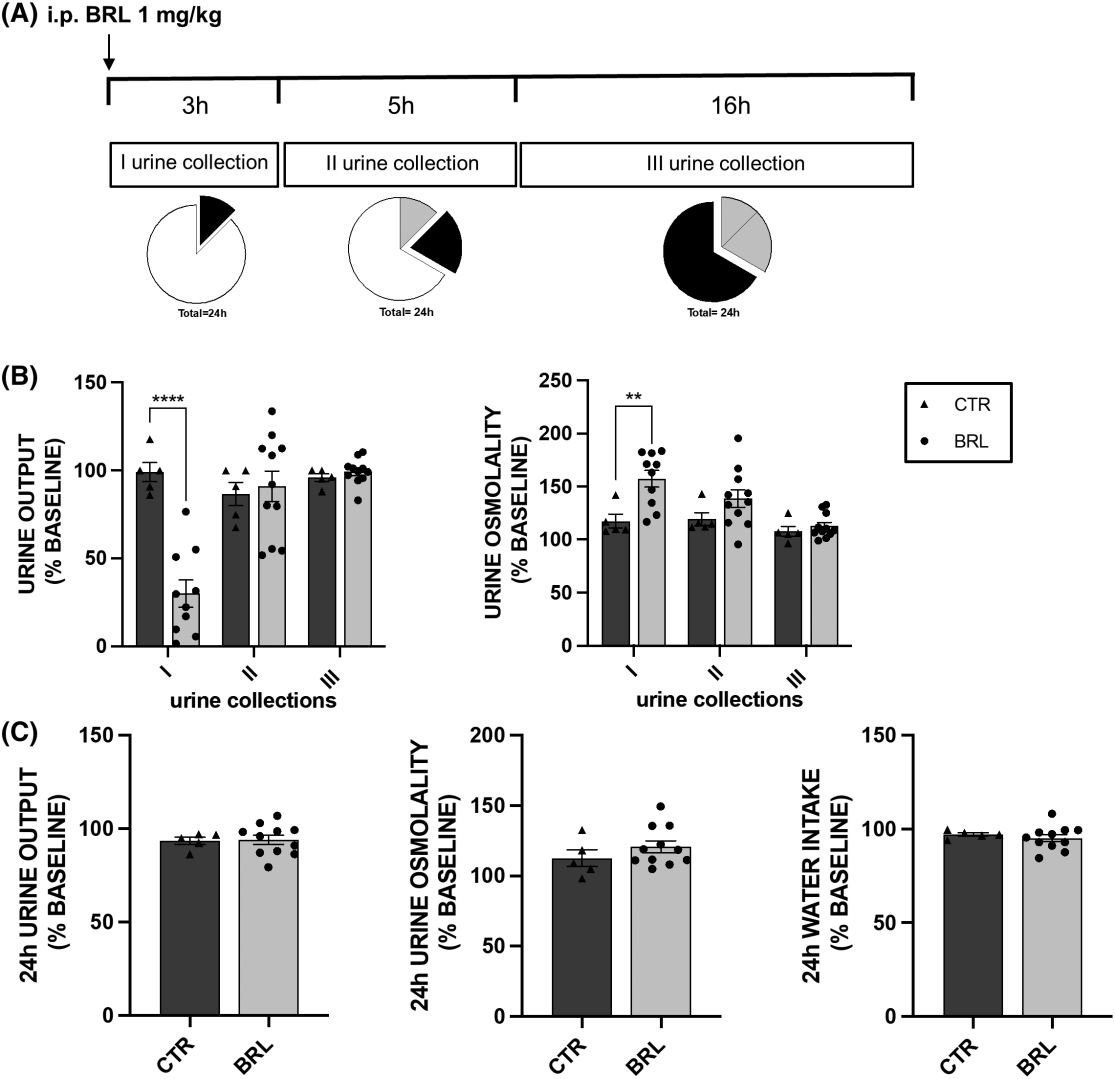
A single i.p. injection of BRL37344 1 mg/kg promoted short‐lasting reabsorption of water and solutes in X‐NDI mice. 16 X‐NDI mice were individually placed in metabolic cages. For 3 days, 11 received a single i.p. injection of BRL37344 1 mg/kg (BRL), whereas 5 control animals received saline alone (CTR) (protocol 1). (A) Urine samples were collected 3 h after injection (I urine collection), 5 h after the I collection (II urine collection) and again 16 h later (III urine collection). The whole pie represents 24 h and each part of whole proportionally indicates the duration of the urine collection time windows. In particular, the black part corresponds to the current urine collection, the grey part to the sample collection already done and white part to the missing collection. In all scatter plots, data were given as mean ± SEM and each dot represents the mean effect of the injection in 3 days experiments on different parameters for each mouse. Data were expressed as a percentage of the mean value measured in 5 days preceding the experiments (baseline set ad 100%, not represented in the plot) for each mouse. The missing dots are due to the lack of urine production by some mice. (B) Urine output and urine osmolality were measured in the three time windows in CTR and BRL X‐NDI mice. CTR mice showed minimal fluctuations around their baseline (set as 100%) for urine output and osmolality analysis. Conversely, BRL37344 i.p. injection reduced urine output (≈70%) and increased urine osmolality (≈40%) during the first 3 h (I). The antidiuretic effect of BRL37344 was not seen in the later urine collections (II, III). Significant differences between CTR and BRL were tested by a two‐tailed unpaired *t*‐test.**p < 0.01, ****p < 0.0001. Significant differences between data measured during the monitoring (baseline) and data collected during the treatment, for both CTR and BRL, tested by two‐tailed paired *t*‐test, were reported in the text. (c) 24 h urine output and osmolality and water intake of CTR and BRL X‐NDI mice. No significant effect was seen in 24 h parameters of BRL mice. For graphical representation and data analysis details see above.

Mice received for 3 days a single i.p. injection of vehicle or BRL37344 1 mg/kg body weight and urine were collected for 3 h after injection (I urine collection), 5 h after the I collection (II collection) and 16 h after the II collection (III collection), as depicted in Figure 1A.

#### 2.1.4 Protocol 2

**FIGURE 2:**
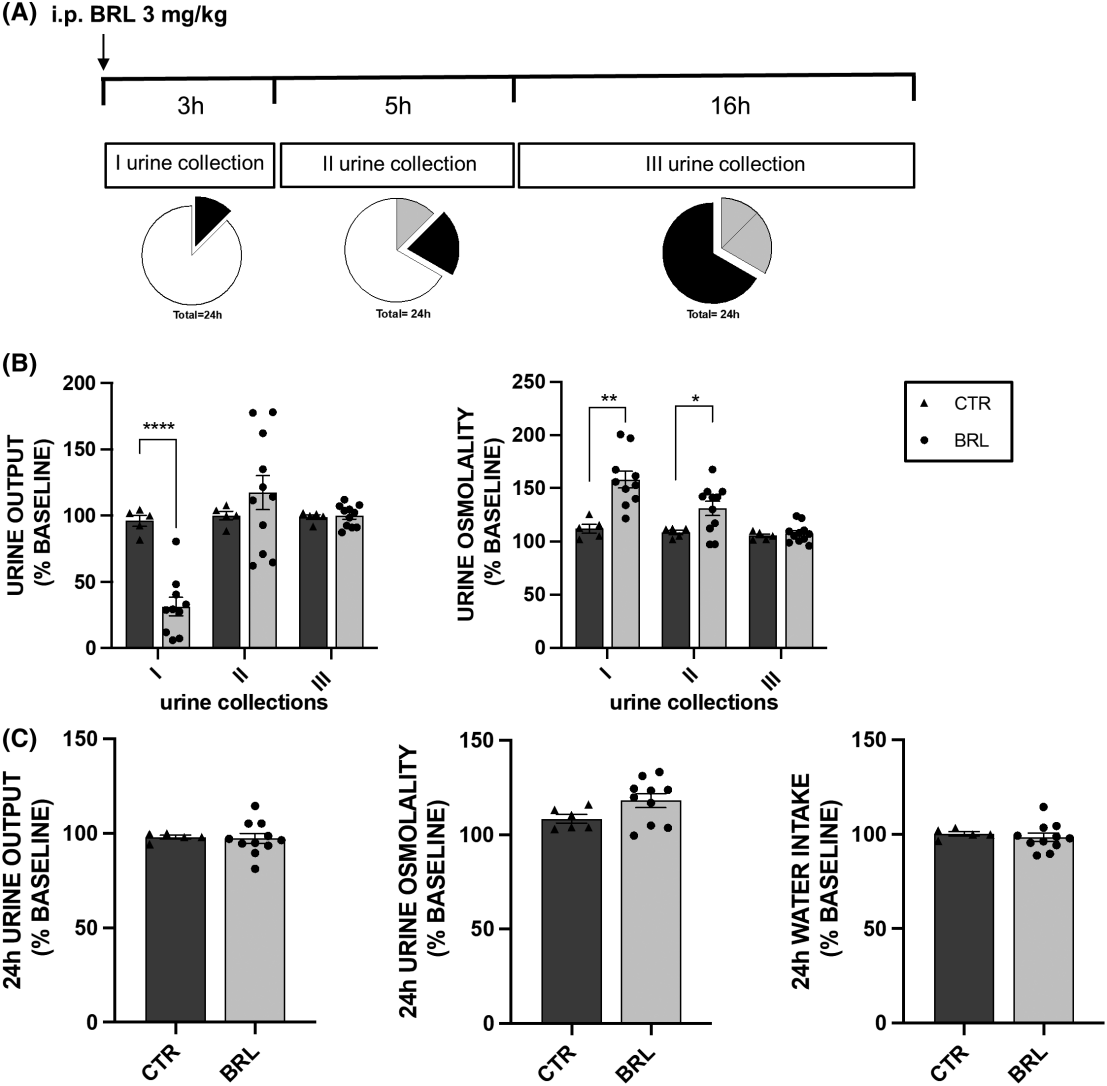
The effect of a single intra‐peritoneal injection of BRL37344 3 mg/kg was similar to that caused by BRL37344 1 mg/kg. 16 X‐NDI mice were individually placed in metabolic cages. For 3 days, 11 received a single i.p. injection of BRL37344 3 mg/kg (BRL), whereas 5 control animals received saline alone (CTR) (protocol 2). (A) Urine samples were collected 3 h after injection (I urine collection), 5 h after the I collection (II urine collection) and again 16 h later (III urine collection). The whole pie represents 24 h and each part of the whole proportionally indicates the duration of the urine collection time windows. In particular, the black part corresponds to the current urine collection, the grey part to the sample collection already done and the white part to the missing collection. All data of urine output and osmolality and water intake were expressed, for each mouse, as a percentage setting the average of values measured in the 5 days preceding the experiment (baseline, not represented in the plot) as 100%. In all scatter plots, data were given as mean ± SEM and each dot represents the mean effect of the injection in 3 days of experiments on different parameters for each mouse. The missing dots were due to the lack of urine production by some mice. (B) Urine output and urine osmolality in the three time windows in CTR and BRL X‐NDI mice. BRL37344 i.p. injection reduced urine output and increased urine osmolality during the first 3 h (I). The antidiuretic effect of BRL37344 was not observed in the subsequent urine collections (II, III). Significant differences between CTR and BRL were tested by two‐tailed unpaired *t*‐test. *p < 0.05, **p < 0.01, ****p < 0.0001. Significant differences between data measured during the monitoring (baseline) and data collected during the treatment, for both CTR and BRL, tested by two‐tailed paired *t*‐test, were reported in the text. (C) 24 h urine output and osmolality and water intake of CTR and BRL X‐NDI mice. No significant effect was seen in 24 h parameters of BRL mice. For graphical representation and data analysis details see above.

Mice received the same treatment described in protocol 1 at 3 mg/kg body weight (see Figure 2A).

#### 2.1.5 Protocol 3

For three consecutive days, mice received 3 i.p. injections of vehicle/BRL37344 1 mg/kg, the second was 3 h after the first and the third 16 h after the second injection (see Figure S1a).

#### 2.1.6 Protocol 4

**FIGURE 3:**
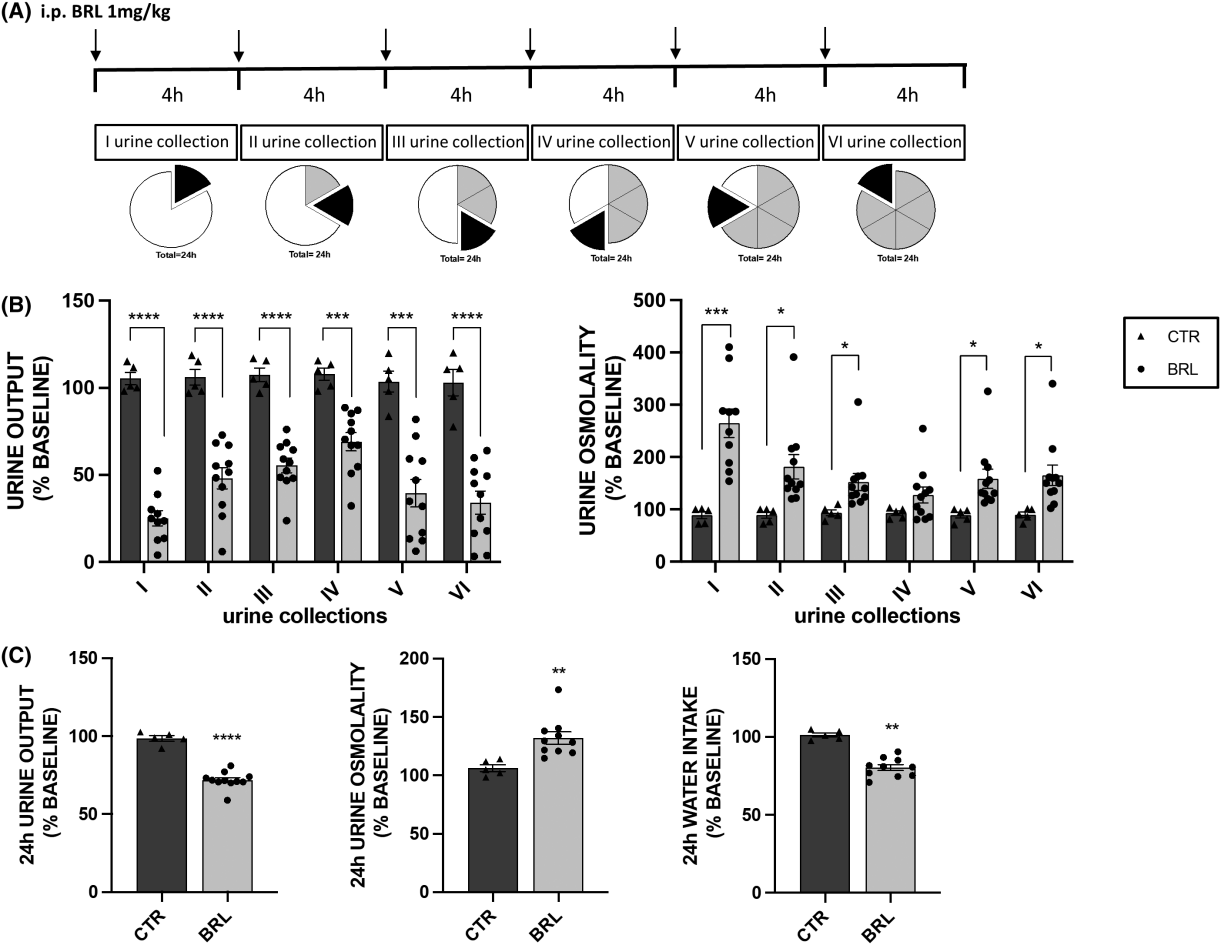
Multiple repeated intra‐peritoneal injections of BRL37344 1 mg/kg improved urine concentrating ability in X‐NDI mice. 16 X‐NDI mice were individually placed in metabolic cages. 11 X‐NDI mice received every 4 h 6 i.p. injections (showed by arrows) of BRL37344 1 mg/kg (BRL), while 5 received saline alone (CTR) (protocol 4). (A) Urine samples were collected every 4 h after injections (I, II, III, IV, V, VI urine collections). The whole pie represents 24 h and each part of the whole proportionally shows the duration of the urine collection time windows. The black part corresponds to the current urine collection, the grey part to the sample collection already done and the white part to the missing collection. All data of urine output, osmolarity and water intake were expressed, for each mouse, as a percentage setting the average of values measured, in the same time windows, in the 5 days preceding the experiment (baseline, not represented in the plot) as 100%. In all scatter plots data were given as mean ± SEM and each dot represents a mouse. The missing dots were due to the lack of urine production by some mice. (B) Urine output and urine osmolality in the six time windows in CTR and BRL X‐NDI mice. All BRL injections significantly reduced urine output and increased urine osmolality compared to vehicle injections in CTR mice. Significant differences between CTR and BRL were tested by two‐tailed unpaired *t*‐test. *p < 0.05, ***p < 0.001 ****p < 0.0001. Significant differences between data measured during the monitoring (baseline) and data collected during the treatment, for both CTR and BRL, tested by two‐tailed paired t‐test, were reported in the text. (C) 24 h urine output and osmolality and water intake of CTR and BRL X‐NDI mice. 6 BRL37344 injections promoted a huge water and solutes reabsorption: 24 h urine output and water intake were reduced by ≈30% and ≈20% respectively and in parallel, 24 h urine osmolality was increased of about ≈30% in BRL mice compared to CTR mice. For graphical representation and data analysis details see above. Significant differences between CTR and BRL were tested by two‐tailed unpaired *t*‐test. **p < 0.01 ****p < 0.0001.

Mice were injected every 4 h for 24 h with vehicle alone or BRL37344 1 mg/kg (see Figure 3A). At the end of this treatment, X‐NDI mice were killed, and kidneys were used in RT‐PCR (4 kidneys from CTR and 8 from BRL mice), in Western blotting (3 kidneys from CTR and 9 from BRL mice) and in immunofluorescence experiments (3 kidneys from CTR and 5 from BRL mice).

### 2.2 Urine and serum chemistry

Blood samples were collected in heparinized tubes by cardiac puncture. Plasma and urine electrolytes were measured using the ion‐selective electrode method. Urine osmolarities were measured using a vapour pressure osmometer (model 5600, Wescor, Logan, UT).

#### 2.2.1 Determination of GFR

GFR of conscious mice, before and after the treatment reported above in protocol 4, was measured by fluorescein isothiocyanate labelled sinistrin clearance after a single retro‐orbital injection and consecutive blood sampling from the tail vein.^23^

#### 2.2.2 Statistics

For statistical analysis, GraphPad Prism software (version 9.4.0, La Jolla, CA) was used. For comparison between two groups, CTR versus BRL, unpaired t‐test (two‐tailed) was used. For comparison of before versus after vehicle/BRL37344 injection paired t‐test (two‐tailed) was performed. *p* < 0.05 was considered statistically significant.

#### 2.2.3 RNA isolation and RT‐quantitative PCR

Total RNA was extracted from kidneys (4 kidneys from CTR and 8 from BRL mice, see protocol 4) using TRIzol (Sigma‐Aldrich, St. Louis, MO) according to the manufacturer's instructions. First‐strand cDNA was synthesized from 2.5 μg of RNA with a superscript VILO cDNA kit (Thermo Fisher Scientific, Waltham, MA). For quantification of NKCC2, NCC and AQP2 mRNA, real‐time PCR was performed in triplicate using the Applied Biosystem StepONE Real‐time PCR system and the following TaqMan GenExpression Assay (Thermo Fisher Scientific): Mm00490213_m1 for mouse NCC, Mm01275821_m1 for mouse NKCC2 and Mm00437575_m1 for mouse AQP2. The mRNA levels were normalized to reference gene mouse glyceraldehyde 3‐phosphate dehydrogenase (Mm 99999915_g1, Thermo Fisher Scientific). Each reaction was carried on as a single plex reaction. The relative quantification of gene expressions was evaluated with the StepONE software according to the comparative Ct method.

#### 2.2.4 Western blotting

The whole kidneys were isolated from X‐NDI mice treated as described in protocol 4 (3 kidneys from CTR and 9 from BRL mice) and homogenized in RIPA buffer.^24^ 30 μg of each lysate was separated by SDS‐PAGE using Mini‐PROTEAN® TGX Stain‐Free™ _Precast Gels Bio‐Rad (Bio‐rad, Hercules, CA) and analysed by Western blotting. After blocking with 3% bovine serum albumin, blots were incubated with specific primary antibodies (see above) and then with horseradish peroxidase–conjugated secondary antibodies. Blots were revealed by Western ECL Substrate (Bio‐Rad) with Chemidoc XRS, equipped with Image Lab 6.1 Software (Bio‐Rad) used for the protein quantification. Each target was normalized to the total protein content using the Stain‐Free™technology^25^ according to the manufacturer's instructions.

#### 2.2.5 Immunofluorescence

Mouse kidneys (3 kidneys from CTR and 5 from BRL mice, see protocol 4) were fixed overnight with 4% paraformaldehyde at 4°C, cryopreserved in 30% sucrose for 24 h and then embedded in optimal cutting temperature medium. Thin cryosections (7 μm) were subjected to immunofluorescence analysis. Antigen retrieval was performed by boiling sections in citrate buffer (10 mM sodium citrate, pH 6). After blocking with 1% bovine serum albumin in PBS for 30 min, sections were incubated with the primary antibodies anti‐NKCC2, anti‐pNKCC2, anti‐NCC, anti‐pNCC, anti‐AQP2, anti‐pAQP2 and the appropriate AlexaFluor‐conjugated secondary antibodies (Thermo Fisher Scientific) according to the manufacturer's instructions. Confocal images were obtained with a confocal laser‐scanning fluorescence microscope (Leica TSC‐SP5, Wetzlar, Germany) using a 63X objective (HCX PL APO CS63.0 Å ~ 1.40 OIL UV).

## 3 RESULTS

### 3.1 A single intra‐peritoneal injection of BRL37344 promotes transient tubular reabsorption of water and solutes in X‐NDI mice

The study included 16 X‐NDI animals, divided into two experimental groups: a control group (CTR, *N* = 5) and a treated group (BRL, *N* = 11). To establish a reliable baseline of urinary parameters (urine output and osmolarity, urine electrolytes) of each animal, urine samples were collected and analysed for 5 days before experimentation in three different time windows: 10 AM–1 PM (3 h), 1 PM–6 PM (5 h) and 6 PM–10 AM (18 h). Water and food consumption was evaluated every 24 h. On experiment days, the animals in the BRL37344 group were given an i.p. injection of BRL37344 1 mg/kg at 10 am, while the CTR group received an i.p. injection of vehicle alone. Urine was collected in the time windows described above (Figure 1A).

Given the intra‐species variability of urinary parameters among animals, each measured value was expressed as a percentage of the mean value of the same parameter measured in the 5 days preceding the experimentation (baseline, set as 100%). Each experiment was repeated for three consecutive days and in the histograms each dot represented the average of the values of that parameter measured over the 3 days.

In histograms 1B and 1C, for each urine collection, one can compare each parameter to the baseline of the same group, and one can also compare the BRL animals to the CTR group. The statistical analysis shown in the figure referred to the latter comparison. According to the histograms in Figure 1B, the CTR animals (dark grey columns) showed minimal, statistically insignificant, percentage fluctuations around their urine output value at baseline.

In contrast, treated animals (light grey columns, BRL) showed a dramatic reduction in urine output (−70%, *p* < 0.0001, paired *t*‐test) in the first collection, compared to their baseline urine output. This reduction was also statistically significant compared to the urine output of the CTR group. Furthermore, in BRL‐treated animals, the osmolarity of the first urine collection was significantly 57% higher compared to their baseline and 35% higher compared to the CTR group of mice.

However, no effect of BRL37344 was seen in the later urine collections (II and III, at 5 and 16 h).

In addition, the analysis of urine electrolyte excretion in Table 1 showed a significant reduction in urinary excretion of Na^+^, K^+^ and Cl^−^ only in the first urine collection from BRL mice compared to that measured in CTR mice.

**TABLE 1:** Effect of 1 i.p. injection of BRL37344 1 mg/kg on urinary electrolytes excretion in X‐NDI mice (protocol 1).

		CTR	1 i.p. BRL 1 mg/kg
I urine collection (CTR = 5 vs. BRL = 10)	uNa+/ucr (mEq/mg)	0.35 ± 0.02	0.15 ± 0.05*
	uK+/ucr (mEq/mg)	0.61 ± 0.11	0.40 ± 0.04*
	uCl−/ucr (mEq/mg)	0.65 ± 0.02	0.27 ± 0.07**
	ucr mg/dL	2.79 ± 0.47	8.11 ± 1.29*
	ucr/urine output (mg)	0.06 ± 0.004	0.05 ± 0.01
II urine collection (CTR = 5 vs. BRL = 11)	uNa+/ucr (mEq/mg)	0.33 ± 0.04	0.24 ± 0.05
	uK+/ucr (mEq/mg)	0.56 ± 0.08	0.68 ± 0.09
	uCl−/ucr (mEq/mg)	0.64 ± 0.02	0.58 ± 0.13
	ucr mg/dL	3.26 ± 0.64	6.17 ± 1.31
	ucr/urine output (mg)	0.10 ± 0.02	0.11 ± 0.01
III urine collection (CTR = 5 vs. BRL = 11)	uNa+/ucr (mEq/mg)	0.66 ± 0.01	0.69 ± 0.03
	uK+/ucr (mEq/mg)	0.90 ± 0.02	0.97 ± 0.03
	uCl−/ucr (mEq/mg)	1.27 ± 0.13	1.25 ± 0.09
	ucr mg/dL	1.76 ± 0.33	2.32 ± 0.33
	ucr/urine output (mg)	0.50 ± 0.02	0.46 ± 0.02
24 h (CTR = 5 vs. BRL = 11)	uNa+/ucr (mEq/mg)	0.64 ± 0.05	0.63 ± 0.02
	uK+/ucr (mEq/mg)	0.86 ± 0.03	0.88 ± 0.02
	uCl−/ucr (mEq/mg)	1.22 ± 0.17	1.10 ± 0.06
	ucr mg/dL	1.82 ± 0.44	2.69 ± 0.41
	ucr/urine output (mg)	0. 61 ± 0.05	0.62 ± 0.01
	Food intake (g)	5.35 ± 0.16	5.24 ± 0.15

*Note*: For 3 days, 11 X‐NDI mice received a single i.p. injection of BRL37344 1 mg/kg (BRL), whereas 5 animals received saline alone (CTR). Urine chemistry measurements were carried out on urine samples collected 3 h after injection (I urine collection), 5 h after the I collection (II urine collection) and 16 h later (III urine collection). Different mice numbers between collections were due to the lack of urine production by some mice. Food consumption was also reported. Data were given as mean ± SEM.

* *p* < 0.05;

** *p* < 0.01.

As shown in Figure 2 and Table 2, the administration of BRL37344 at a higher dose (3 mg/kg), keeping the experimental design unchanged, did not produce a stronger or more lasting antidiuretic effect compared to the 1 mg/kg dose. The comparison of electrolyte excretion at baseline and following injection of vehicle or BRL37344 (1 or 3 mg/kg) is reported in Table S1.

**TABLE 2:** Effect of 1 i.p. injection of BRL37344 3 mg/kg on urinary electrolytes excretion in X‐NDI mice (protocol 2).

		CTR	1 i.p. BRL 3 mg/kg
I urine collection (CTR = 5 vs. BRL = 10)	uNa+/ucr (mEq/mg)	0.31 ± 0.04	0.12 ± 0.02***
	uK+/ucr (mEq/mg)	0.56 ± 0.04	0.35 ± 0.03**
	uCl−/ucr (mEq/mg)	0.75 ± 0.13	0.27 ± 0.04***
	ucr mg/dL	2.70 ± 0.68	7.01 ± 0.96*
	ucr/urine output (mg)	0.06 ± 0.01	0.04 ± 0.01
II urine collection (CTR = 5 vs. BRL = 11)	uNa+/ucr (mEq/mg)	0.32 ± 0.04	0.25 ± 0.04
	uK+/ucr (mEq/mg)	0.65 ± 0.07	0.74 ± 0.10
	uCl−/ucr (mEq/mg)	0.93 ± 0.06	0.76 ± 0.07
	ucr mg/dL	2.74 ± 0.40	5.17 ± 1.33
	ucr/urine output (mg)	0.10 ± 0.01	0.12 ± 0.01
III urine collection (CTR = 5 vs. BRL = 11)	uNa+/ucr (mEq/mg)	0.74 ± 0.04	0.80 ± 0.03
	uK+/ucr (mEq/mg)	1.0 ± 0.04	0.92 ± 0.02
	uCl−/ucr (mEq/mg)	1.70 ± 0.26	1.40 ± 0.06
	ucr mg/dL	1.53 ± 0.38	2.24 ± 0.28
	ucr/urine output (mg)	0.45 ± 0.01	0.45 ± 0.01
24 h (CTR = 5 vs. BRL = 11)	uNa+/ucr (mEq/mg)	0.65 ± 0.01	0.62 ± 0.02
	uK+/ucr (mEq/mg)	0.91 ± 0.02	0.85 ± 0.03
	uCl−/ucr (mEq/mg)	1.45 ± 0.17	1.18 ± 0.07
	ucr mg/dL	1.73 ± 0.36	2.67 ± 0.37
	ucr/urine output (mg)	0.62 ± 0.01	0.65 ± 0.03
	Food intake (g)	5.38 ± 0.14	5.26 ± 0.11

*Note*: Urine chemistry measurements were carried out on urine samples collected 3 h after the injection of BRL37344 3 mg/kg or vehicle (I urine collection), 5 h after the I collection (II urine collection) and again 16 h later (III urine collection). Different mice numbers between collections were due to the lack of urine production by some mice. Food consumption was also measured. The experiment was repeated three times. Data were given as mean ± SEM.

* *p* < 0.05;

** *p* < 0.01;

*** *p* < 0.001 (CTR = 5, BRL = 11).

This finding suggested that the 1 mg/kg dose of BRL37344 was the minimal effective dose to obtain antidiuretic effect in X‐NDI mice. Therefore, all next experiments were carried out using this dose of BRL37344.

### 3.2 Repeated intra‐peritoneal injections of BRL37344 over 24 h improve urine concentrating ability in X‐NDI mice

The effect of three, 3 h‐spaced BRL37344 injection 1 mg/kg is reported in Supplementary Results (Supplementary Figure S1 and Table S2, S3).

Next, we subjected the X‐NDI mice to 6 i.p. of BRL37344 (or vehicle alone), spaced 4 h apart, to maintain a sustained antidiuretic effect throughout the 24 h. No side effects on the viability or behaviour of the mice were observed. Six urine collections were analysed. As expected, the histograms in Figure 3B show that the urine output of the CTR animals did not significantly change compared to their baseline values, while that of the BRL group was dramatically reduced, both compared to their baseline value and compared to the volumes measured in the CTR group. In BRL‐treated animals, the first urine collection confirmed a significant reduction in urine output of about −76% and an increase in urine osmolarity, almost +200%, compared to CTR mice. Strikingly, the later urine collections, each every 4 h, confirmed a sustained antidiuretic effect, with urine output remaining significantly reduced (by −55%, −48%, −36%, −62% and −67% respectively), and urine osmolarity remaining significantly elevated (by +103%, +63%, +37%, +78%, and + 83%, respectively) compared to the CTR group of animals. Overall, the 24 h cumulative urine output (Figure 3C) was significantly reduced by 27% and urine osmolarity increased by 25% by BRL37344 compared to CTR mice. Interestingly, the reduction in diuresis was accompanied by a significant reduction (−20%) in water intake in the BRL group. The graphical representation of row data before and after injections of each mouse, presented in supplementary figure (S2), provides compelling evidence of the antidiuretic activity of BRL37344, irrespective of the severity of the polyuric/hyposthenuric phenotype.

**FIGURE 4:**
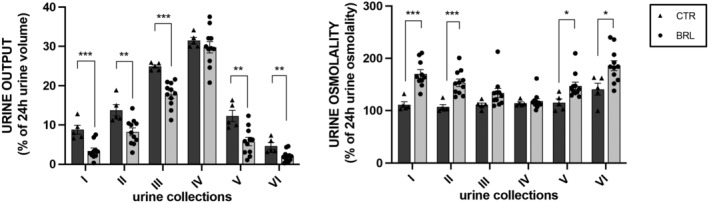
Multiple repeated intra‐peritoneal injections of BRL37344 1 mg/kg (protocol 4) did not change X‐NDI mice's habits. To exclude the possible side effects of multiple BRL injections on the physiological activities of animals (drowsiness, lethargy, malaise, etc.), we compared the urine volume and osmolality of each collection, expressed as a percentage of urine output and osmolality of 24 h (100%), of CTR with those from BRL mice. Even though at each collection, except for the fourth, the urine volume produced by BRL‐treated mice was always statistically reduced and urine osmolality always increased compared to CTR mice, the urine production at each collection followed the same trend between CTR and BRL mice, with a peak in the night hours (10 PM–2 AM, IV collection) when the animals were more active. Significant differences between CTR and BRL were tested by two‐tailed unpaired *t*‐test. Data were given as mean ± SEM and each dot represents a mouse. *p < 0.05, **p < 0.01, ***p < 0.001.

In Figure 4 we plotted the volume of each urine collection as a percentage of the cumulative urine output volume in 24 h (100%). It is interesting to note that urine output in each collection follows the same daily pattern between CTR and BRL groups, with a peak in the night hours (10 PM–2 AM, IV collection), likely when the animals are more active. However, in each collection, except for the fourth, the volume produced by BRL‐treated animals was always statistically reduced compared to control animals.

The analysis of urine electrolytes in CTR and BRL mice is reported in Table 3. The comparison of electrolyte excretion at baseline and following 6 injections of vehicle or BRL37344 (1 mg/kg) is reported in Supplementary Table S4.

**TABLE 3:** Effect of 6 i.p. injections of BRL37344 1 mg/kg on urinary electrolytes excretion in X‐NDI mice (protocol 4).

		CTR	6 i.p. BRL 1 mg/kg
I urine collection after I i.p. (CTR = 5 vs. BRL = 10)	uNa+/ucr (mEq/mg)	0.33 ± 0.05	0.17 ± 0.04*
	uK+/ucr (mEq/mg)	0.74 ± 0.06	0.38 ± 0.04***
	uCl−/ucr (mEq/mg)	0.60 ± 0.04	0.24 ± 0.04****
	ucr mg/dL	2.27 ± 0.63	6.56 ± 1.13*
	ucr/urine output (mg)	0.09 ± 0.01	0.07 ± 0.01
II urine collection after II i.p. (CTR = 5 vs. BRL = 11)	uNa+/ucr (mEq/mg)	0.38 ± 0.09	0.19 ± 0.02*
	uK+/ucr (mEq/mg)	0.78 ± 0.11	0.53 ± 0.06*
	uCl−/ucr (mEq/mg)	0.72 ± 0.14	0.37 ± 0.04**
	ucr mg/dL	2.43 ± 0.70	5.02 ± 1.21
	ucr/urine output (mg)	0.13 ± 0.004	0.12 ± 0.01
III urine collection after III i.p. (CTR = 5 vs. BRL = 11)	uNa+/ucr (mEq/mg)	0.75 ± 0.05	0.52 ± 0.05*
	uK+/ucr (mEq/mg)	1.30 ± 0.05	0.98 ± 0.05**
	uCl−/ucr (mEq/mg)	1.40 ± 0.13	1.05 ± 0.07*
	ucr mg/dL	1.18 ± 0.30	2.29 ± 0.41
	ucr/urine output (mg)	0.12 ± 0.01	0.13 ± 0.01
IV urine collection after IV i.p. (CTR = 5 vs. BRL = 11)	uNa+/ucr (mEq/mg)	0.94 ± 0.15	0.97 ± 0.06
	uK+/ucr (mEq/mg)	0.96 ± 0.05	0.92 ± 0.03
	uCl−/ucr (mEq/mg)	1.28 ± 0.23	1.23 ± 0.08
	ucr mg/dL	1.35 ± 0.38	1.88 ± 0.22
	ucr/urine output (mg)	0.17 ± 0.02	0.18 ± 0.01
V urine collection after V i.p. (CTR = 5 vs. BRL = 11)	uNa+/ucr (mEq/mg)	0.73 ± 0.04	0.58 ± 0.06
	uK+/ucr (mEq/mg)	0.90 ± 0.09	0.49 ± 0.03****
	uCl−/ucr (mEq/mg)	1.02 ± 0.11	0.75 ± 0.07
	ucr mg/dL	1.63 ± 0.43	3.33 ± 0.64
	ucr/urine output (mg)	0.08 ± 0.02	0.06 ± 0.01
VI urine collection after VI i.p. (CTR = 5 vs. BRL = 11)	uNa+/ucr (mEq/mg)	0.72 ± 0.31	0.43 ± 0.04
	uK+/ucr (mEq/mg)	0.56 ± 0.10	0.37 ± 0.03*
	uCl−/ucr (mEq/mg)	0.86 ± 0.30	0.61 ± 0.05
	ucr mg/dL	2.69 ± 0.90	4.67 ± 0.66
	ucr/urine output (mg)	0.04 ± 0.002	0.04 ± 0.004
24 h (CTR = 5 vs. BRL = 11)	uNa+/ucr (mEq/mg)	0.77 ± 0.13	0.57 ± 0.03
	uK+/ucr (mEq/mg)	1.10 ± 0.16	0.76 ± 0.04*
	uCl−/ucr (mEq/mg)	1.34 ± 0.27	0.90 ± 0.06*
	ucr mg/dL	1.45 ± 0.48	2.60 ± 0.35
	ucr/urine output (mg)	0.55 ± 0.07	0.59 ± 0.03
	Food intake (g)	5.10 ± 0.30	4.86 ± 0.14
Plasma (CTR = 5 vs. BRL = 11)	Na+ (mEq/mL)	137.3 ± 6.24	146.5 ± 2.10
	K+ (mEq/L)	9.13 ± 0.88	7.30 ± 0.72
	Cl– (mEq/L)	109.5 ± 9.74	120.8 ± 3.43
	cr (mg/dL)	0.18 ± 0.01	0.20 ± 0.01
	GFR (μL/min)	293 ± 8	305 ± 9

*Note*: 11 X‐NDI mice received 6 i.p. injections, spaced 4 h apart, of BRL37344 1 mg/kg (BRL), whereas 5 were injected with saline alone (CTR). Urine samples were collected every 4 h after injections (I, II, III, IV, V, VI urine collections). Sodium, potassium, chloride and creatinine were measured either in spot urine (I, II, III collections) or in cumulative urine samples (24 h). Different mice numbers between collections were due to the lack of urine production by some mice. Plasma chemistry measurements were carried out at the end of the experiment for both groups. GFR measurements were performed after 6 i.p. injections in conscious CTR and BRL mice. Data were given as mean ± SEM.

* *p* < 0.05;

** *p* < 0.01;

*** *p* < 0.001;

^****^ *p* < 0.0001.

Urinary excretion of Na^+^, K^+^ and Cl^−^, normalized for creatinine, was strongly reduced in BRL‐treated animals in the I, II and III urine collections, strongly suggesting that the BRL37344 treatment promoted tubular salts reabsorption. Urinary excretion of Na^+^, K^+^ and Cl^−^ in the IV collection was not affected by BRL37344 treatment. At later collections, urine electrolyte excretion showed a declining trend in BRL compared to CTR mice although the differences were not always statistically significant. The overall analysis of the urine electrolytes excretion in the 24 h confirmed that multiple injections of BRL37344 promoted both salutes and water tubular reabsorption. Instead, neither the plasma concentration of the same electrolytes nor the GFR were affected by BRL37344 treatment compared to CTR mice. Measurement of GFR 1 h after the first injection of BRL37344 revealed no significant differences compared to control mice (BRL 301 μL/min ± 7 vs. CTR 298 μL/min ± 5), indicating that BRL37344 did not acutely alter GFR at this time point. These findings suggest that the reduced urine output observed following repeated BRL37344 injections may involve mechanisms other than changes in GFR immediately after administration. Importantly, no significant differences in food intake were observed between the two groups indicating that the reduction of both urine output and urine electrolytes excretion was not due to decreased food consumption.

Of note, the same experiment was performed in β3‐AR knockout mice^26^ which did not show changes in urine parameters after repeated BRL37344 injections (see Supplemental Figure S3).

### 3.3 Effect of multiple repeated intra‐peritoneal injections of BRL37344 on renal transporters in X‐NDI mice

The effect of BRL37344 on urine volume and composition prompted us to investigate the possible effects of BRL37344 treatment on the transcription, protein abundance, phosphorylation and subcellular localization of NKCC2, NCC and AQP2 in the kidneys of animals injected 6 times in 24 h with vehicle alone or BRL37344.

**FIGURE 5:**
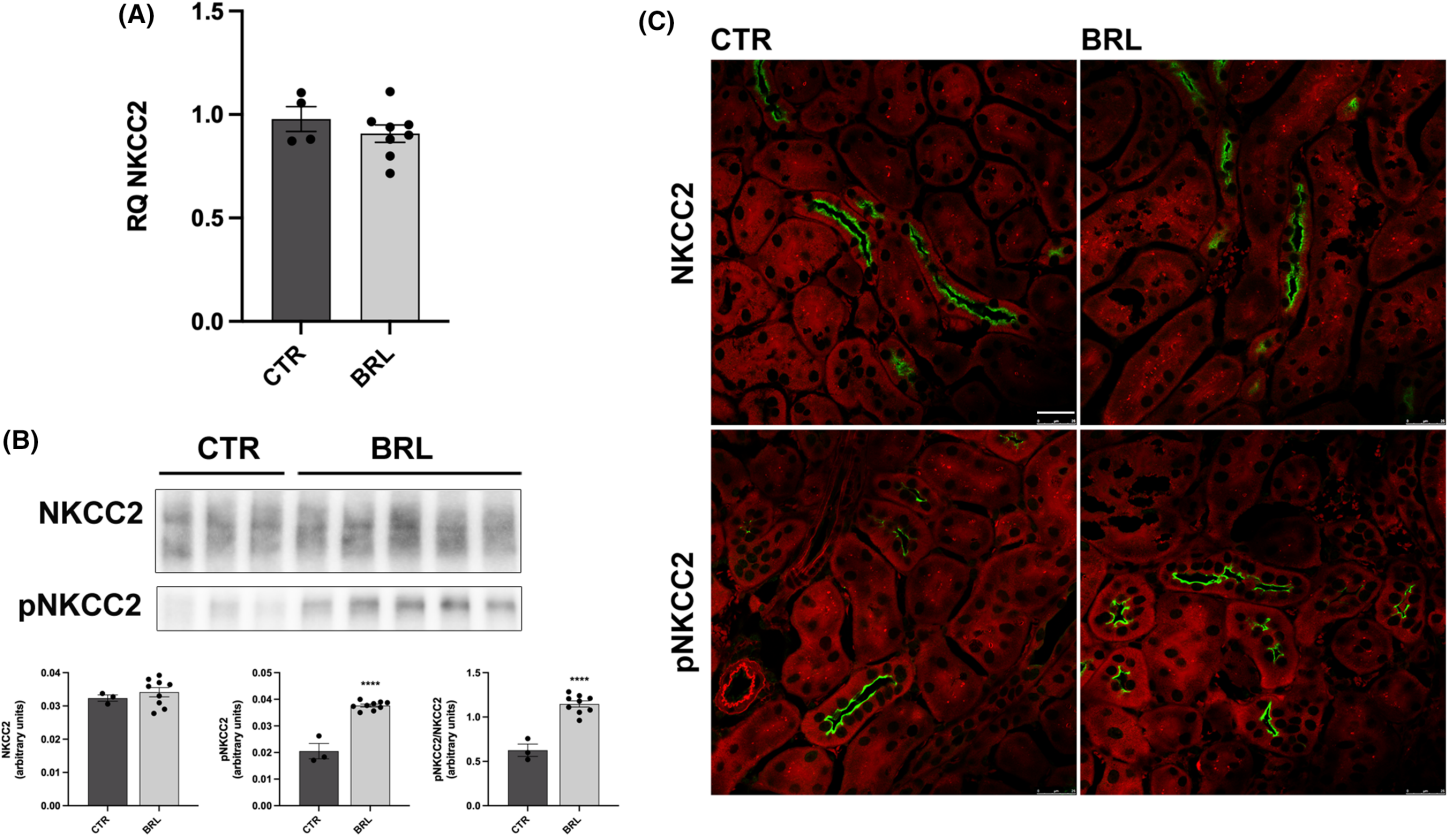
Multiple repeated BRL37344 1 mg/kg i.p. injections promoted NKCC2 activation in the thick ascending limb of X‐NDI mice. (A) At the end of the 6 i.p injections/24 h experiment, a quantitative reverse transcription polymerase chain reaction was performed on kidneys from X‐NDI treated with saline (CTR, *n* = 4) or with BRL37344 (BRL, *n* = 8). Relative quantification of gene expression (RQ) was performed setting the amount of NKCC2 mRNA in CTR as 1. No differences were seen in NKCC2 transcription between the two groups. The experiment was repeated three times and comparable results were obtained. In the scatter plot data were given as mean ± SEM and each dot represents the average of data from three experiments for each mouse. (B) Western blotting with anti‐NKCC2 and antiphosphorylated NKCC2 antibodies was carried out using homogenates prepared from whole kidneys of CTR (*n* = 3) and BRL (*n* = 9) mice. Representative lanes were reported in the figure. The expression levels of each protein were normalized to total protein content using Stain‐free™ gels technologies. Densitometric analysis showed a two‐fold increase in pNKCC2 (active form), normalized to total NKCC2, in BRL compared to CTR mice. No differences were seen in NKCC2 abundance. The experiment was repeated three times and comparable results were obtained. In the scatter plot, data were given as mean ± SEM and each dot represents the average of data from three experiments for each mouse. ****p < 0.0001 with two‐tailed unpaired Student's t‐test (C) Kidneys from CTR and BRL mice were subjected to immunofluorescence localization of NKCC2 (in green) and pNKCC2 (in green) and counterstained with Evans blue (red) (CTR = 3, BRL = 5). The number of NKCC2‐positive TAL cells and the localization of NKCC2 were similar in two groups but the fluorescence intensity of pNKCC2 was increased in BRL mice. Representative images were shown. The experiment was repeated three times and comparable results were obtained. (bar = 25 μm).

**FIGURE 6:**
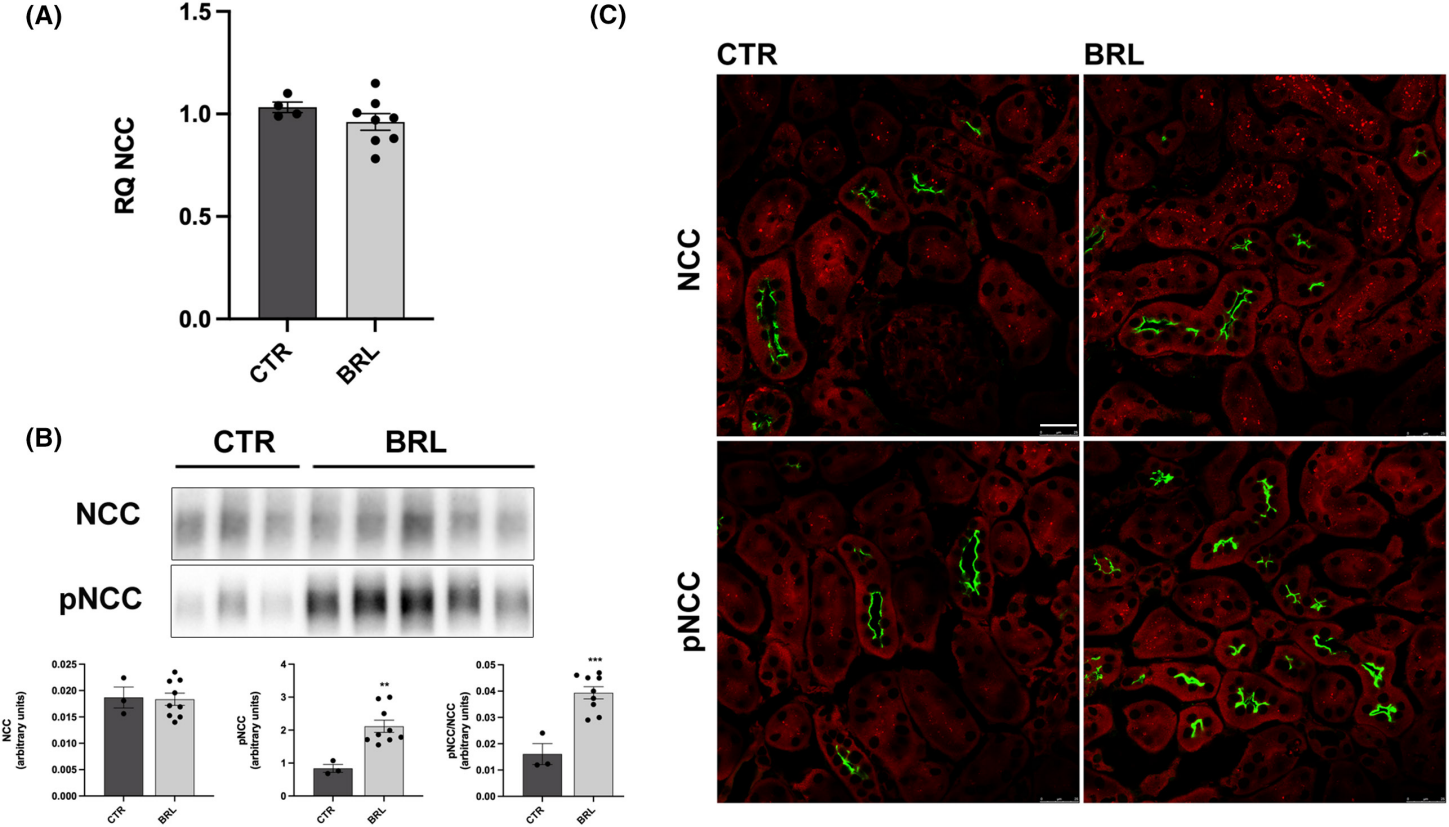
Multiple repeated BRL37344 1 mg/kg i.p. injections promoted NCC activation in the distal convolute tubule of X‐NDI mice. (A) After 6 i.p injections/24 h, quantitative reverse transcription polymerase chain reaction was performed on kidneys from X‐NDI treated with saline (CTR, *n* = 4) or with BRL37344 (BRL, *n* = 8). NCC relative quantification indicated that the treatment with BRL did not affect NCC transcription. Comparable results were obtained from three independent experiments. In the scatter, plot data were given as mean ± SEM and each dot represents the average of data from three experiments for each mouse. (B) Western blotting analysis of total and phosphorylated forms of NCC on total kidney homogenates from CTR (*n* = 3) and BRL (*n* = 9) mice revealed strong activation of NCC (pNCC) in BRL mice with no differences in NCC abundance between the two groups. Representative lanes were reported in the figure. Densitometric analysis showed the expression levels of NCC and pNCC normalized to total protein content using Stain‐free™ gels technologies. The experiment was repeated three times and comparable results were obtained. In the scatter plot data were given as mean ± SEM and each dot represents the average of data from three experiments for each mouse. **p < 0.01, ***p < 0.001 with two‐tailed unpaired Student's *t*‐test. (C) kidney sections from CTR (*n* = 3) and BRL (*n* = 5) mice, counterstained with Evans Blue (in red), were subjected to immunofluorescence localization of NCC and pNCC (in green). BRL injections did not change the expression levels and localization of NCC but increased pNCC apical expression in DCT cells. The experiment was repeated three times and comparable results were obtained. (bar = 25 μm).

As shown in Figure 5A, BRL37344 did not alter NKCC2 transcript levels, as shown by real‐time PCR experiments. The abundance of NKCC2, measured by immunoblotting, was also unchanged, but the levels of phosphorylated NKCC2, normalized for the total form, were increased by 255% (Figure 5B). These data were also confirmed by immunofluorescence analysis on kidney sections of CTR or treated mice. In Figure 5C the apical localization and the fluorescent intensity of total NKCC2 were apparently unchanged between CTR and BRL mice. However, the fluorescent intensity and the number of pNKCC2 reactive tubules were increased in BRL‐treated mice, compared to CTR. Figure 6 shows the same analysis for NCC in the DCT. In analogy with NKCC2, NCC transcript and total protein levels were unaffected by BRL37344 treatment, although the phosphorylated form of NCC was increased, as shown by immunoblotting (+250%) and immunofluorescence with anti‐pNCC antibody.

**FIGURE 7:**
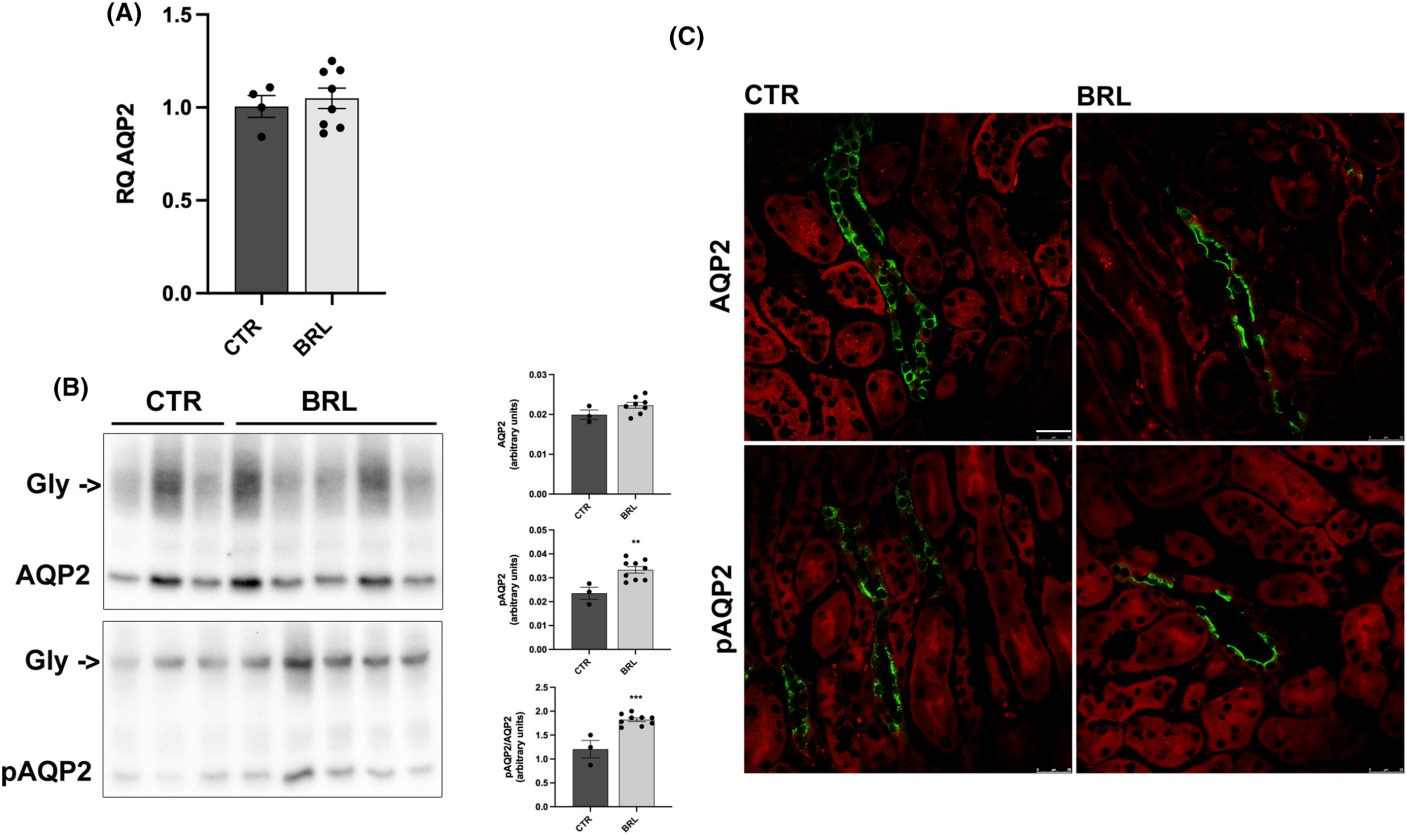
Multiple repeated intra‐peritoneal injections of BRL37344 1 mg/kg promoted AQP2 phosphorylation at Ser256 increasing its expression at the apical plasma membrane of principal cells in the collecting ducts of X‐NDI mice. (A) Real‐time RT‐PCR indicated that 6 i.p. injections of BRL37344 did not change renal AQP2 gene transcription levels (CTR = 4, BRL = 8). Comparable results were obtained from three independent experiments. In the scatter plot, data were given as mean ± SEM and each dot represents the average of data from three experiments for each mouse. (B) Western blotting analysis showed no statistically significant differences in total AQP2 but a clear increase of AQP2 phosphorylation at Ser256 in total kidney lysates BRL (*n* = 9) versus CTR mice (*n* = 3). Densitometric analysis showed the expression levels of AQP2 and pAQP2 normalized to total protein content using Stain‐free™ gels technologies. Comparable results were obtained from three independent experiments. In the scatter plot, data were given as mean ± SEM and each dot represents the average of data from three experiments for each mouse. **p < 0.01, ***p < 0.001 with two‐tailed unpaired Student's *t*‐test. (C) Kidney sections from CTR (*n* = 3) and BRL (*n* = 5) mice, counterstained with Evans Blue (in red), were subjected to immunofluorescence localization of AQP2 and pAQP2 (in green). BRL injections clearly increased AQP2 phosphorylation and promoted its accumulation at the apical membrane of collecting ducts principal cells. The experiment was repeated three times and comparable results were obtained (bar = 25 μm).

As for AQP2, Figure 7 shows that BRL37344 did not increase the abundance of AQP2 mRNA (Figure 7A) and the protein abundance of total AQP2 (Figure 7B). However, BRL37344 treatment increased the abundance of pAQP2 (at Ser256) by approximately 50% (Figure 7B, pAQP2) and immunofluorescence showed an increase in the fluorescence signal and a prominent apical translocation of phosphorylated AQP2 (Figure 7C).

## 4 DISCUSSION

X‐linked nephrogenic diabetes insipidus is a rare congenital disease still missing a real cure.^5^ Current approaches for the treatment of congenital NDI aim to limit the urine output rather than acting at a causative level bypassing the inactivation of the mutated AVPR2 in these patients. Among the different therapeutic approaches proposed over the years, one of the most promising is to identify other GPCRs in AVPR2‐positive renal cells, the stimulation of which activates the cAMP pathway. Studies in this framework evaluated the efficacy of stimulating the secretin receptor (SCTR),^22^ the calcitonin receptor (CTR)^27^ and the prostaglandin receptors^21^ as antidiuresis triggers in NDI animal models.

We previously identified and characterized the role of the β3‐AR receptor in renal cells.^17^ The β3‐AR appears to be an ideal candidate to vicariate unfunctional mutated AVPR2. Indeed, it is a receptor expressed in a limited number of tissues,^28^, ^29^, ^30^ is expressed in most of the kidney tubular cells expressing the AVPR2, likewise the AVPR2 is coupled to the cAMP pathway, and is resistant to agonist‐induced desensitization,^31^, ^32^ including renal cells.^33^ All these features suggest that, in addition to hypothesizing a possible role in regulating the water and salt balance in particular physiological conditions, pharmacological stimulation of β3‐AR in the kidney could represent a successful treatment to cure X‐NDI. The present study provides compelling evidence that continuous stimulation of the β3‐AR receptor can produce a long‐lasting antidiuretic effect in the experimental mouse model of X‐linked nephrogenic diabetes insipidus.^21^ In these animals, it has been demonstrated that AVPR2 deletion results in downregulation of the AQP2 and AQP3 protein levels with unchanged levels of the NKCC2 and NCC co‐transporters and ENaC channel.^21^ Here we tested the effect of β3‐AR selective stimulation by BRL37344 on urine output and osmolarity, urine electrolytes excretion and water intake, using X‐NDI mice in metabolic cages.

The animals, all extremely polyuric, showed a certain variability in urine output, so we not only compared BRL37344 treated animals with vehicle‐injected animals but also the renal phenotype of each mouse with their baseline recorded in the days preceding the treatment. This is to exclude the contribution of environmental factors or stress related to the handling of the animals during injection, which could alter the release of endogenous catecholamines on the β3‐ARs. In both comparisons, BRL37344 showed a dramatic antidiuretic effect, importantly, without affecting the GFR, neither after a single injection nor at the end of 6 injections. In support of this, in a previous work^17^ we had already demonstrated that BRL37344 did not alter GFR measured at 1, 2, 3 and 4 h after an i.p. injection of BRL37344 into wt mice. Collectively, our analysis across multiple studies reaffirms that BRL37344 exerts a potent antidiuretic effect without altering GFR, as demonstrated in both X‐NDI animals and wt mice.

The effect of a single i.p. administration of BRL37344 1 mg/kg produced a 70% reduction in urine output within 3 h after administration. This effect was accompanied by an increase in urine osmolarity and a decrease in urinary Na^+^, K^+^ and Cl^−^ excretion. This suggests that the antidiuretic effect of BRL37344 is mediated through an increase in water and electrolyte reabsorption in the renal tubules. Interestingly, a second injection of BRL37344, 3 h after the first, produced the same effects in the following 3 h, and a third injection, 3 h after the second, showed a significant, although attenuated, effect in the urine collection of the 18 h following the injection (see supplementary results, Figure S1). These results suggest that the antidiuretic effect of a single i.p. injection of BRL37344 is maximal within 3 h of administration but at longer times the molecule tends to lose effectiveness. For this reason, we injected the X‐NDI mice 6 times, at 4 h intervals, to verify the effect, in the whole 24 h, on all urinary parameters. This mimics the administration of a slow‐release BRL37344 preparation. Compared to their baseline, all mice produced a significantly smaller volume of more hypertonic urine in all urine collections except for the fourth collection. Most likely, the explanation for the lack of antidiuretic effect of the BRL37344 in that time window is that from 10 PM to 2 AM animals are in the most active phase of their daily cycle, they feed and drink more and this could determine osmotic diuresis, on which the BRL37344 has no effect. Nonetheless, the overall 24 h effect of continuous treatment with BRL37344 was a 27% decrease in urine output, an increase in urinary osmolarity by 25%, and a 20% decrease in water intake. We could speculate that treatment with BRL37344, coupled with a low‐sodium and low‐protein diet, the latter generally prescribed for X‐NDI patients, limiting the osmotic diuresis, could potentially enhance the efficacy of the BRL37344 treatment and provide greater benefits on X‐NDI phenotype.

Although BRL37344 produced a clear increase in urine osmolality in X‐NDI mice, analysis of urinary excretion of Na^+^, K^+^ and Cl^−^, normalized for urinary creatinine, showed that treatment with BRL37344 reduces renal excretion of these electrolytes. Urinary creatinine excretion represents a reliable normalization factor for assessing changes in water and electrolyte excretion induced by BRL37344 treatment. Despite alterations in water and electrolyte excretion rates, urinary creatinine excretion remains unaffected (see ucr/urine output in all Tables**)**, providing a stable reference point for normalization. Instead, a reduced urinary excretion of electrolytes was particularly evident following the first three administrations of BRL37344 and visible in the cumulative 24 h collection. This suggests that BRL37344 activates the main tubular ion transporters responsible for the genesis and maintenance of the corticomedullary osmotic gradient which represents the driving force that allows the osmotic reabsorption of water in the CD during antidiuresis. We focused our analyses on the apical transporters in the TAL and DCT, both expressing β3‐AR.^18^, ^34^ Molecular analysis of the kidneys of experimental mice indicates that BRL37344 did not increase the expression of NKCC2 and NCC, which are not downregulated in the X‐NDI model. Rather, it increases their phosphorylated/activated status in the luminal membrane of the TAL and DCT, respectively. The picture that emerges from these observations is that treatment with BRL37344 activates antidiuresis by acting at several levels: it increases the tubular reabsorption of electrolytes, favouring the formation of the corticomedullary osmotic gradient, and it also increases the apical translocation of pAQP2, favouring the reabsorption of water on the CD. By demonstrating the effectiveness of BRL37344 in reducing urine output in all treated X‐NDI animals, irrespective of their baseline urine output/osmolality, we underscore its therapeutic potential in ameliorating the polyuria associated with this disease.

An important strength of the approach we propose, which could favour clinical studies in patients with X‐NDI, is the fact that mirabegron, a specific human β3‐AR agonist, is already approved for clinical use in the treatment of overactive bladder (OAB).^28^, ^34^, ^35^, ^36^, ^37^, ^38^, ^39^ We recently demonstrated in OAB patients that chronic treatment with mirabegron for 12 weeks increases urinary excretion of AQP2 and NKCC2, an indirect but reliable index of plasma membrane expression of these two proteins.^40^ The main limitation of the study concerns the lack of pharmacokinetic studies of the compound BRL37344 in the animal model used. However, the pharmacokinetics of mirabegron, the β3AR agonist that we hypothesize may have application in the treatment of X‐NDI in humans, is known.

It is worth noting that the safe dose of mirabegron for use in humans is 50–100 mg/day, close to the 1 mg/kg dose, we used for BRL37344 in mice. This might suggest that the results obtained in the present study on X‐NDI mice may have translational relevance to human clinical practice on X‐NDI patients. If confirmed in clinical trials, this finding could lead to the development of new treatments for this rare genetic condition, which currently has no cure.

## AUTHOR CONTRIBUTIONS

**Serena Milano:** Conceptualization (equal); data curation (equal); formal analysis (equal); methodology (equal); validation (equal); visualization (equal); writing – review and editing (equal). **Ilenia Saponara:** Data curation (equal); investigation (equal); methodology (equal); software (equal); validation (equal). **Andrea Gerbino:** Data curation (equal); formal analysis (equal); methodology (equal); validation (equal); writing – review and editing (equal). **Monica Carmosino:** Conceptualization (equal); investigation (equal); supervision (equal); writing – review and editing (equal). **Maria Svelto:** Conceptualization (equal); funding acquisition (equal); supervision (equal); writing – review and editing (equal). **Giuseppe Procino:** Conceptualization (equal); funding acquisition (equal); investigation (equal); resources (equal); supervision (lead); writing – original draft (lead); writing – review and editing (equal).

## FUNDING INFORMATION

This research was funded by the Italian MUR: ‘Proof of Conceptl, grant number POC01_00072 to GP and ‘Attraction and International Mobility’ PON R&I 2014–2020, Azione I.2 (code AIM1893457) to SM. This research was also funded by Fondazione TELETHON (grant number GGP15083 to MS).

## CONFLICT OF INTEREST STATEMENT

The authors declare that the research was conducted in the absence of any commercial or financial relationships that could be construed as a potential conflict of interest.

## DISCLOSURE STATEMENT

The authors have nothing to disclose.

## Supporting information


**Data S1:** xxxx.

## Data Availability

The data that supports the findings of this study are available in the supplementary material of this article.
