# Supplementary material for: The β3‐AR agonist BRL37344 ameliorates the main symptoms of X‐linked nephrogenic diabetes insipidus in the mouse model of the disease
Source: J Cell Mol Med. 2024 Apr 23;28(8):e18301. doi: 10.1111/jcmm.18301 (PMC11037407; doi:10.1111/jcmm.18301)
Supplement: Supplementary file 1 — Data S1: xxxx. [file JCMM-28-e18301-s001.docx]

**SUPPLEMENTARY MATERIALS**

**Supplementary Results**

**Three repeated intraperitoneal injections of BRL37344 promote a more durable reabsorption of water and solutes in X-NDI mice.**

We investigated whether administering the drug in three separate BRL injections (1mg/kg), spaced three hours apart, could produce a more sustained antidiuretic effect over the 24h, thus mimicking the effect of a slow-release BRL formulation.

For three consecutive days, mice were injected with BRL37344 or vehicle alone at 10am, 1pm and 4pm, and urine collected in the following time windows: 10am-1pm (3h), 1pm-4pm (3h), 4pm-10am (18h), as shown in Fig. S1a. In the histogram in Fig. S1b, the urine output of the vehicle-injected animals (dark gray columns, CTR) was comparable to their baseline. In BRL-injected animals, as expected, in the I urine collection, the urine output was reduced by 73% (p<0.0001), the volume collected 3h after the second BRL booster (II urine collection) was also significantly reduced by 64% (p<0.0001), and also the urine output collected during the 18h following the 3rd injection was significantly reduced, although only by 14% (p<0.0001) compared to the baseline of the same animals. The reduction was also statistically significant compared with CTR animals, as reported in the figure. Urine osmolarity after BRL treatment was also increased in each urine collections, with a more clear effect in the first two (+26% and +40%, respectively) and smaller (+6%), although statistically significant, in the urine of the third collection. A comparison between the two groups was reported in the figure. Analyzing the cumulative urine collection over the 24h (Fig. S1c), the urine output of the BRL group was reduced by about 17% compared to their baseline, and 20% compared to the CTR group. Urine osmolarity was 8% increased (p<0.01) in the BRL group compared to their baseline, and 17% compared to the CTR mice. Interestingly, the partial correction of polyuria also reflected in a significant 17% reduction in the water intake in the BRL group, both if compared with their baseline (p<0.0001) and with CTR group.

Table S2 reports the analysis of urine electrolytes in CTR and BRL mice after repeated injections. Although urine osmolarity was increased by BRL treatment in all urine collections, urinary excretion of Na+, K+ and Cl-, normalized for creatinine, was significantly reduced in BRL-treated animals, compared to CTR animals, in the first two urine collections, suggesting that the drug not only promoted water reabsorption, but also tubular reabsorption of salts.

In the cumulative 24h urine collection there were no statistically significant differences in the urinary excretion of Na+, K+, Cl- and creatinine, nor in the food intake. Furthermore, the lack of significant fluctuations in the urinary excretion of solutes in the CTR group (vehicle-injected), compared to their baseline (see supplemental table S3), confirms that the observed effects were specific to the BRL treatment.


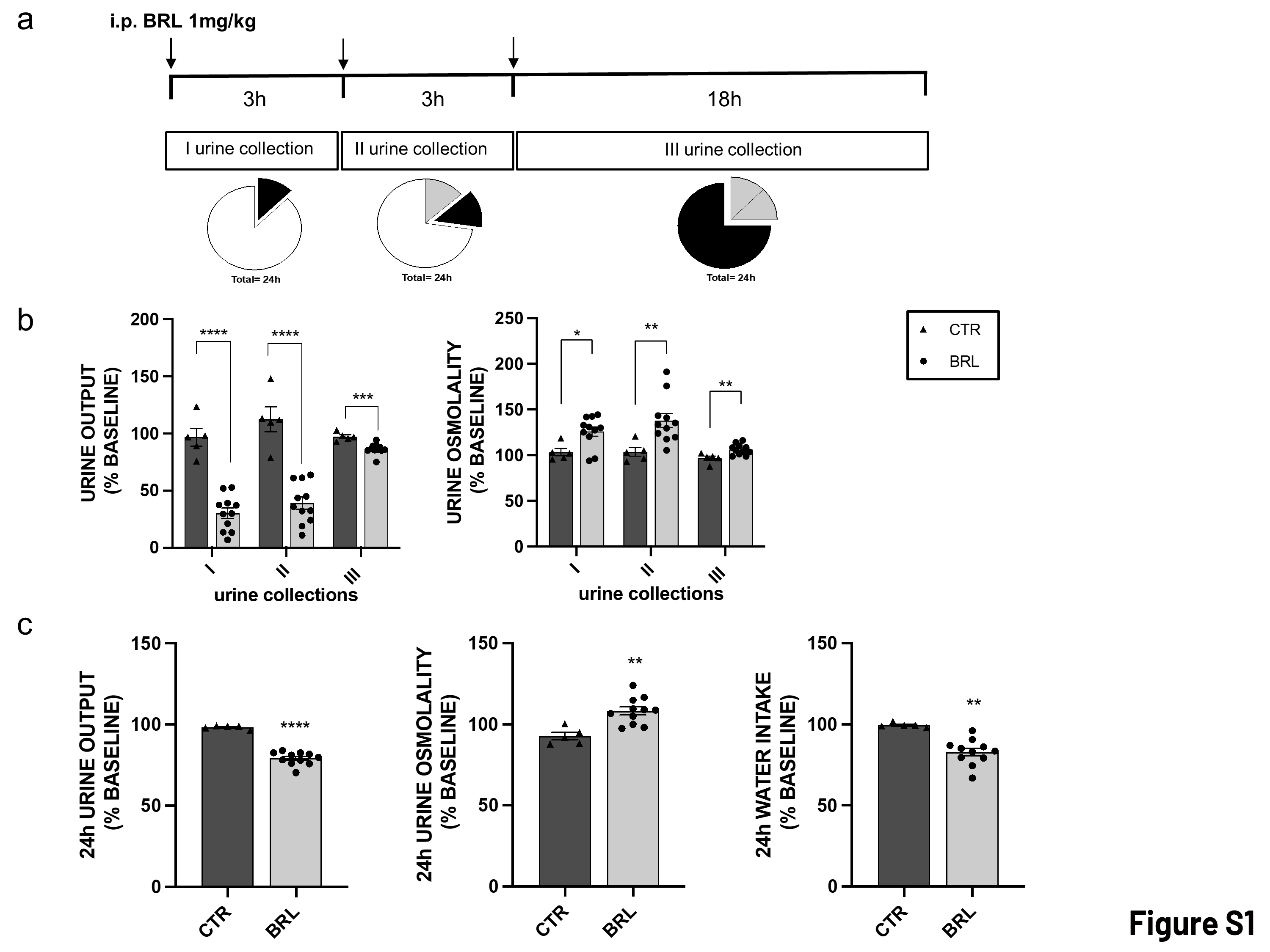


**Supplementary Figure 1. Three repeated intraperitoneal injections of BRL37344 1mg/kg promoted a long-lasting reabsorption of water and solutes in X-NDI mice.**

16 X-NDI mice were individually placed in metabolic cages. For 3 days, 11 X-NDI mice received 3 i.p. injection (indicated by arrows) of BRL37344 1 mg/kg (BRL), whereas 5 received saline alone (CTR). (a) Urine samples were collected 3 hours after the first injection (I urine collection), 3 hours after the second injection (II urine collection) and 18 hours the last injection (III urine collection). The whole pie represents 24 hours and each part of the whole proportionally shows the duration of the urine collection time windows. Black part corresponds to the current urine collection, gray part to the sample collection already done and white part to the missing collection. All data of urine output and osmolality and water intake were expressed, for each mouse, as a percentage setting the average of values measured, in the same time windows, in the 5 days preceding the experiment (baseline, not represented in the plot) as 100%. In all plots, data were given as mean ± SEM and each dot represents the mean effect in three days experiments of inections on different parameters for each mouse. The missing dots were due to the lack of urine production by some mice. (b) Urine output and urine osmolality in the three time windows in CTR and BRL X-NDI mice. The first and second BRL37344 injections reduced urine output of about 70% and increased urine osmolality of about 20% and 40%, respectively. The antidiuretic effect of the third BRL37344 injection, even though smaller than that observed after the first 2 injections (I and II collections), was statistically significant. Significant differences between CTR and BRL were tested by two-tailed unpaired t-test. *P<0.05, **P<0.01, ***P<0.001 ****P<0.0001. Significant differences between data measured during the monitoring (baseline) and data collected during the treatment, for both CTR and BRL, tested by two-tailed paired t-test, were reported in the text. (c) 24h urine output and osmolality and water intake of CTR and BRL X-NDI mice. 3 BRL37344 injections significantly reduced 24h urine output (≈20%) and consequently 24h water intake (≈17%) and, in parallel, increased 24h urine osmolality (≈17%) in BRL mice compared to CTR mice. For graphical representation and data analysis details see above. Significant differences between CTR and BRL were tested by two-tailed unpaired t-test. **P<0.01 ****P<0.0001.

**
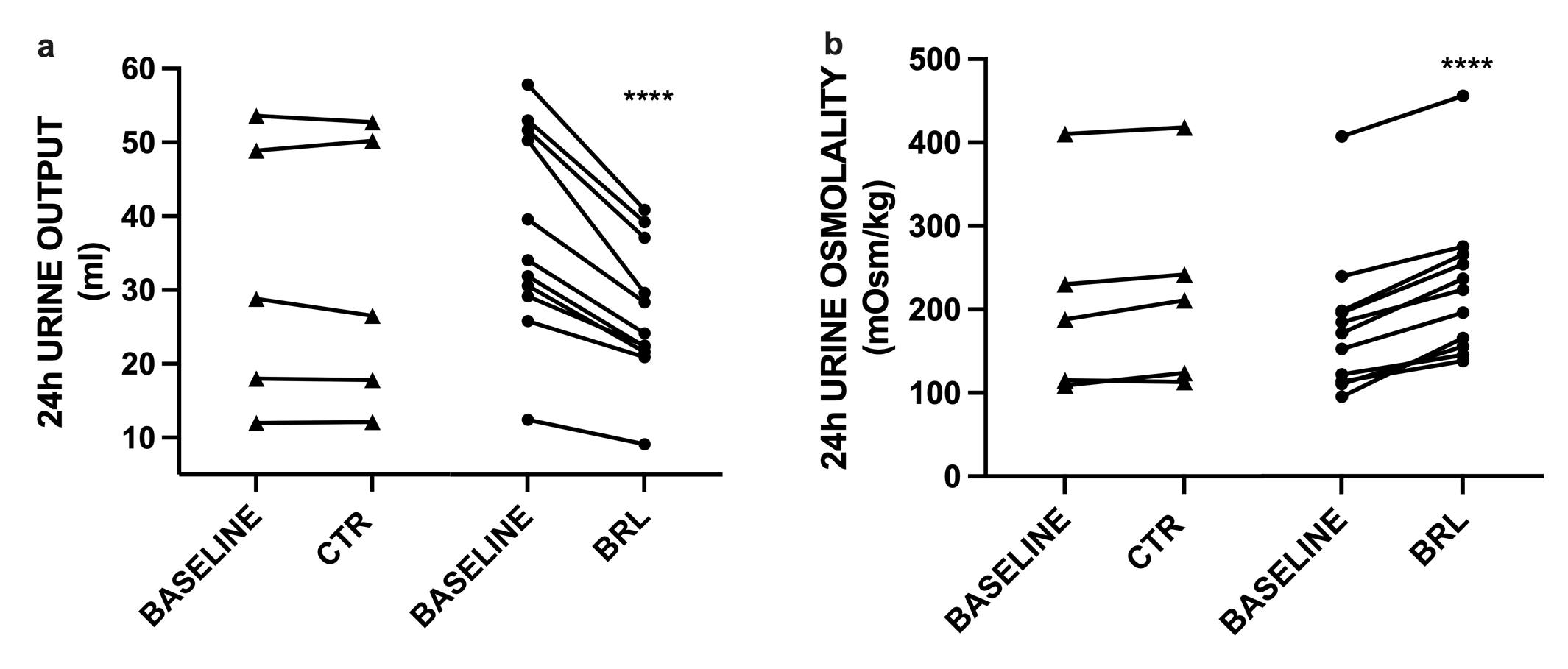
**

**Supplementary S2. Multiple repeated intraperitoneal injections of BRL37344 1mg/kg improved urine concentrating ability in X-NDI mice: row data of urine output and osmolality (mean values ± SEM were reported in Fig. 3).**

Sixteen X-NDI mice were individually housed in metabolic cages. Eleven X-NDI mice received intraperitoneal injections of BRL37344 at a dose of 1 mg/kg every 4 hours for 24 hours (BRL group), while five mice received saline injections alone (CTR group) according to protocol 4. The plots display raw data of 24-hour urine output (a) and urine osmolality (b). Mean values ± SEM, expressed as % of the baseline werereported in Figure 3. Administration of six BRL37344 injections significantly decreased 24-hour urine output and increased urine osmolality in all X-NDI mice, regardless of the severity of their polyuria. Paired t-tests were conducted to compare data measured during the baseline monitoring period with data collected after injections (saline or BRL37344) for both CTR and BRL groups. ****P<0.0001.

**
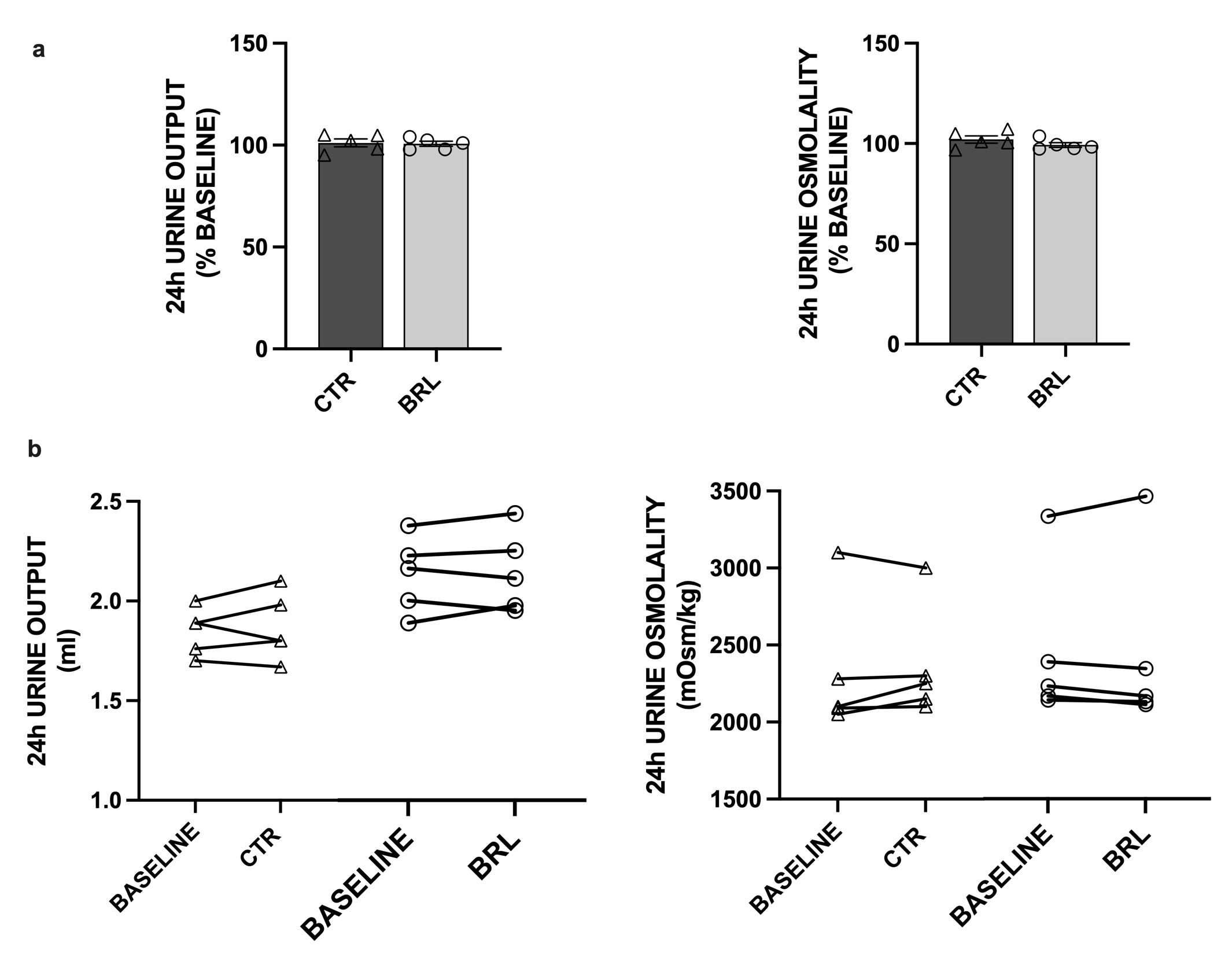
**

**Supplementary S3. Multiple repeated intraperitoneal injections of BRL37344 1mg/kg did not change urinary parameters in β3-AR knockout mice.**

Ten male β3-AR knockout mice [^1^](#_ENREF_1) were divided into two experimental groups: control (CTR) and BRL37344-treated (BRL) groups, 5 mice in each group. Mice were individually housed in metabolic cages, and every 4 hours for 24 hours, they received intraperitoneal injections of BRL37344 at a dose of 1 mg/kg (BRL group) or vehicle alone (CTR group). Twenty-four-hour urine output and osmolality were measured and reported in the figure.

**(a)** The data for urine output and osmolality are expressed as a percentage change relative to the average values measured in the 5 days preceding the experiment (baseline), with baseline values set as 100%. **(b)** The plots display the raw data of urine output and osmolality for each mouse before (baseline) and after 6 injections. As expected, BRL37344 injections did not alter urine output or urine osmolality in β3-AR knockout mice, either when comparing the two experimental groups or when comparing BRL-treated mice before and after 6 intraperitoneal injections. Significant differences between CTR and BRL groups were tested using a two-tailed unpaired t-test. Additionally, significant differences between data measured during the baseline monitoring period and data collected after treatment were tested using a two-tailed paired t-test.

|  |  | **CTR** | | | **BRL** | | |
| --- | --- | --- | --- | --- | --- | --- | --- |
|  |  | **BASELINE** | **1 i.p. vehicle (protocol 1)** | **1 i.p. vehicle (protocol 2)** | **BASELINE** | **1 i.p. BRL 1mg/kg (protocol 1)** | **1 i.p. BRL 3mg/kg (protocol 2)** |
| **I urine collection (CTR=5; BRL =10)** | **uNa+/ucr (mEq/mg)** | 0.33 ± 0.05 | 0.35 ± 0.02 | 0.31 ± 0.04 | 0.37 ± 0.04 | 0.15 ± 0.05 ** | 0.12 ± 0.02 *** |
|  | **uK+/ucr (mEq/mg)** | 0.62 ± 0.05 | 0.61 ± 0.11 | 0.56 ± 0.04 | 0.65 ± 0.06 | 0.40 ± 0.04 ** | 0.35 ± 0.03 *** |
|  | **uCl-/ucr (mEq/mg)** | 0.69 ± 0.04 | 0.65 ± 0.02 | 0.75 ± 0.13 | 0.59 ± 0.07 | 0.27 ± 0.07 ** | 0.27 ± 0.04 *** |
|  | **ucr mg/dl** | 2.33 ± 0.40 | 2.79 ± 0.47 | 2.70 ± 0.68 | 3.39 ± 0.49 | 8.11 ± 1.29 ** | 7.01 ± 0.96 ** |
|  | **ucr/urine output (mg)** | 0.06 ± 0.004 | 0.06 ± 0.004 | 0.06 ± 0.01 | 0.06 ± 0.003 | 0.05 ± 0.01 | 0.04 ± 0.01 |
| **II urine collection (CTR=5; BRL =11)** | **uNa+/ucr (mEq/mg)** | 0.29 ± 0.02 | 0.33 ± 0.04 | 0.32 ± 0.04 | 0.30 ± 0.04 | 0.24 ± 0.05 | 0.25 ± 0.04 |
|  | **uK+/ucr (mEq/mg)** | 0.66 ± 0.04 | 0.56 ± 0.08 | 0.65 ± 0.07 | 0.70 ± 0.03 | 0.68 ± 0.09 | 0.74 ± 0.10 |
|  | **uCl-/ucr (mEq/mg)** | 0.71 ± 0.05 | 0.64 ± 0.02 | 0.93 ± 0.06 | 0.63 ± 0.07 | 0.58 ± 0.13 | 0.76 ± 0.07 |
|  | **ucr mg/dl** | 3.09 ± 0.65 | 3.26 ± 0.64 | 2.74 ± 0.40 | 4.68 ± 0.56 | 6.17 ± 1.31 | 5.17 ± 1.33 |
|  | **ucr/urine output (mg)** | 0.11 ± 0.01 | 0.10 ± 0.02 | 0.10 ± 0.01 | 0.12 ± 0.01 | 0.11 ± 0.01 | 0.12 ± 0.01 |
| **III urine collection (CTR=5; BRL =11)** | **uNa+/ucr (mEq/mg)** | 0.70 ± 0.02 | 0.66 ± 0.01 | 0.74 ± 0.04 | 0.75 ± 0.02 | 0.69 ± 0.03 | 0.80 ± 0.03 |
|  | **uK+/ucr (mEq/mg)** | 1.02 ± 0.06 | 0.90 ± 0.02 | 1.0 ± 0.04 | 0.98 ± 0.06 | 0.97 ± 0.03 | 0.92 ± 0.02 |
|  | **uCl-/ucr (mEq/mg)** | 1.52 ± 0.09 | 1.27 ± 0.13 | 1.70 ± 0.26 | 1.32 ± 0.07 | 1.25 ± 0.09 | 1.40 ± 0.06 |
|  | **ucr mg/dl** | 1.68 ± 0.27 | 1.76 ± 0.33 | 1.53 ± 0.38 | 2.52 ± 0.6 | 2.32 ± 0.33 | 2.24 ± 0.28 |
|  | **ucr/urine output (mg)** | 0.45 ± 0.01 | 0.50 ± 0.02 | 0.45 ± 0.01 | 0.45 ± 0.03 | 0.46 ± 0.02 | 0.45 ± 0.01 |
| **24h (CTR=5; BRL =11)** | **uNa+/ucr (mEq/mg)** | 0.61 ± 0.03 | 0.64 ± 0.05 | 0.65 ± 0.01 | 0.60 ± 0.01 | 0.63 ± 0.02 | 0.62 ± 0.02 |
|  | **uK+/ucr (mEq/mg)** | 0.81 ± 0.05 | 0.86 ± 0.03 | 0.91 ± 0.02 | 0.82 ± 0.04 | 0.88 ± 0.02 | 0.85 ± 0.03 |
|  | **uCl-/ucr (mEq/mg)** | 1.12 ± 0.02 | 1.22 ± 0.17 | 1.45 ± 0.17 | 1.15 ± 0.03 | 1.10 ± 0.06 | 1.18 ± 0.07 |
|  | **ucr mg/dl** | 1.78 ± 0.10 | 1.82 ± 0.44 | 1.73 ± 0.36 | 2.63 ± 0.34 | 2.69 ± 0.41 | 2.67 ± 0.37 |
|  | **ucr/urine output (mg)** | 0.65 ± 0.07 | 0. 61 ± 0.05 | 0.62 ± 0.01 | 0.64 ± 0.02 | 0.62 ± 0.01 | 0.65 ± 0.03 |
|  | **food intake (g)** | 5.21 ± 0.20 | 5.35 ± 0.16 | 5.38 ± 0.14 | 5.1 ± 0.17 | 5.24 ± 0.15 | 5.26 ± 0.11 |

**SUPPLEMENTARY TABLE S1**

**Supplementary Table S1. Comparison of urine electrolytes excretion and food intake before the experiment (baseline) and during the experiment days (1 i.p. injection of BRL37344 1 or 3 mg/kg or vehicle; protocol 1 and 2) for CTR and BRL groups.** Data were presented as mean ± SEM.**P<0.01, ***P<0.001, ****P<0.0001. Significance was determined using paired *t* test for each group. The analysis revealed that CTR and BRL mice did not show significant differences in urine chemistry and food consumption during the monitoring before the experiment (baseline) and it did not change after vehicle injection in CTR mice.

|  |  | |  |  |
| --- | --- | --- | --- | --- |
|  |  | | **CTR** | **3 i.p. BRL**  **1 mg/kg** |
| **I urine collection after I i.p. (CTR=5 vs BRL=11)** | | **uNa+/ucr (mEq/mg)** | 0.30 ± 0.06 | 0.14 ± 0.02 * |
|  |  | **uK+/ucr (mEq/mg)** | 0.62 ± 0.08 | 0.33 ± 0.02 *** |
|  |  | **uCl-/ucr (mEq/mg)** | 0.46 ± 0.13 | 0.15 ± 0.02 ** |
|  |  | **ucr mg/dl** | 2.40 ± 0.74 | 6.86 ± 0.75 ** |
|  |  | **ucr/urine output (mg)** | 0.06 ± 0.002 | 0.05 ± 0.01 |
| **II urine collection after II i.p. (CTR=5 vs BRL=11)** | | **uNa+/ucr (mEq/mg)** | 0.40 ± 0.04 | 0.12 ± 0.02 **** |
|  |  | **uK+/ucr (mEq/mg)** | 0.76 ± 0.05 | 0.44 ± 0.07 * |
|  |  | **uCl-/ucr (mEq/mg)** | 0.74 ± 0.09 | 0.20 ± 0.05 **** |
|  |  | **ucr mg/dl** | 1.90 ± 0.53 | 6.40 ± 1.15 * |
|  |  | **ucr/urine output (mg)** | 0.06 ± 0.01 | 0.06 ± 0.01 |
| **III urine collection after III i.p. (CTR=5 vs BRL=11)** | **uNa+/ucr (mEq/mg)** | | 0.58 ± 0.04 | 0.68 ± 0.03 |
|  | **uK+/ucr (mEq/mg)** | | 0.92 ± 0.04 | 0.95 ± 0.04 |
|  | **uCl-/ucr (mEq/mg)** | | 1.05 ± 0.04 | 1.12 ± 0.04 |
|  | **ucr mg/dl** | | 1.47 ± 0.30 | 2.15 ± 0.30 |
|  | **ucr/urine output (mg)** | | 0.50 ± 0.02 | 0.51 ± 0.02 |
| **24h (CTR=5 vs BRL=11)** | **uNa+/ucr (mEq/mg)** | | 0.56 ± 0.04 | 0.60 ± 0.03 |
|  | **uK+/ucr (mEq/mg)** | | 0.89 ± 0.07 | 0.84 ± 0.02 |
|  | **uCl-/ucr (mEq/mg)** | | 1.08 ± 0.09 | 0.94 ± 0.03 |
|  | **ucr mg/dl** | | 1.60 ± 0.27 | 2.57 ± 0.36 |
|  | **ucr/urine output (mg)** | | 0.64 ± 0.02 | 0.65 ± 0.02 |
|  | **food intake (g)** | | 5.38 ± 0.14 | 5.27 ± 0.11 |

**SUPPLEMENTARY TABLE S2**

**Supplementary Table S2 Effect of 3 i.p. injection of BRL37344 1 mg/kg on urinary electrolytes excretion in X-NDI mice (protocol 3).**

For 3 days, 11 X-NDI mice received 3 i.p. injections of BRL37344 1 mg/kg (BRL), whereas 5 were injected with saline (CTR). Urine samples were collected 3 hours after the first injection (I urine collection), 3 hours after the second injection (II urine collection) and 18 hours after the last injection (III urine collection). Sodium, potassium, chloride and creatinine were measured either in extemporaneous urine (I, II, III collections) or in cumulative urine sample (24h). Daily food intake was also analyzed. Data were given as mean ± SEM. *P<0.05, **P<0.01, ***P<0.001, ****P<0.0001.

|  |  | **CTR** | | **BRL** | |
| --- | --- | --- | --- | --- | --- |
|  |  | **BASELINE** | **3 i.p. Vehicle**  **(protocol 3)** | **BASELINE** | **3 i.p. BRL 1 mg/kg**  **(protocol 3)** |
| **I urine collection after I i.p. (CTR=5; BRL =11)** | **uNa+/ucr (mEq/mg)** | 0.32 ± 0.04 | 0.30 ± 0.06 | 0.26 ± 0.03 | 0.14 ± 0.02 ** |
|  | **uK+/ucr (mEq/mg)** | 0.72 ± 0.07 | 0.62 ± 0.08 | 0.61 ± 0.04 | 0.33 ± 0.02 **** |
|  | **uCl-/ucr (mEq/mg)** | 0.54 ± 0.1 | 0.46 ± 0.13 | 0.38 ± 0.04 | 0.15 ± 0.02 **** |
|  | **ucr mg/dl** | 2.32 ± 0.71 | 2.40 ± 0.74 | 3.78 ± 0.65 | 6.86 ± 0.75 ** |
|  | **ucr/urine output (mg)** | 0.06 ± 0.001 | 0.06 ± 0.002 | 0.06 ± 0.05 | 0.05 ± 0.01 |
| **II urine collection after II i.p. (CTR=5; BRL =11)** | **uNa+/ucr (mEq/mg)** | 0.46 ± 0.08 | 0.40 ± 0.04 | 0.36 ± 0.04 | 0.12 ± 0.02 **** |
|  | **uK+/ucr (mEq/mg)** | 0.81 ± 0.12 | 0.76 ± 0.05 | 0.93 ± 0.06 | 0.44 ± 0.07 **** |
|  | **uCl-/ucr (mEq/mg)** | 0.80 ± 0.17 | 0.74 ± 0.09 | 0.64 ± 0.07 | 0.20 ± 0.05 **** |
|  | **ucr mg/dl** | 2.05 ± 0.71 | 1.90 ± 0.53 | 2.79 ± 0.65 | 6.40 ± 1.15 *** |
|  | **ucr/urine output (mg)** | 0.05 ± 0.002 | 0.06 ± 0.01 | 0.06 ± 0.03 | 0.06 ± 0.01 |
| **III urine collection after III i.p. (CTR=5; BRL =11)** | **uNa+/ucr (mEq/mg)** | 0.62 ± 0.06 | 0.58 ± 0.04 | 0.66 ± 0.03 | 0.68 ± 0.03 |
|  | **uK+/ucr (mEq/mg)** | 1.01 ± 0.05 | 0.92 ± 0.04 | 0.92 ± 0.03 | 0.95 ± 0.04 |
|  | **uCl-/ucr (mEq/mg)** | 1.10 ± 0.12 | 1.05 ± 0.04 | 1.1 ± 0.05 | 1.12 ± 0.04 |
|  | **ucr mg/dl** | 1.43 ± 0.35 | 1.47 ± 0.30 | 1.87 ± 0.32 | 2.15 ± 0.30 |
|  | **ucr/urine output (mg)** | 0.49 ± 0.03 | 0.50 ± 0.02 | 0.53 ± 0.02 | 0.51 ± 0.02 |
| **24h (CTR=5; BRL =11)** | **uNa+/ucr (mEq/mg)** | 0.66 ± 0.04 | 0.56 ± 0.04 | 0.65 ± 0.02 | 0.60 ± 0.03 |
|  | **uK+/ucr (mEq/mg)** | 0.90 ± 0.03 | 0.89 ± 0.07 | 0.89 ± 0.02 | 0.84 ± 0.02 |
|  | **uCl-/ucr (mEq/mg)** | 0.93 ± 0.09 | 1.08 ± 0.09 | 0.87 ± 0.04 | 0.94 ± 0.03 |
|  | **ucr mg/dl** | 1.63 ± 0.40 | 1.60 ± 0.27 | 2.39 ± 0.36 | 2.57 ± 0.36 |
|  | **ucr/urine output (mg)** | 0.65 ± 0.02 | 0.64 ± 0.02 | 0.70 ± 0.02 | 0.65 ± 0.02 |
|  | **food intake (g)** | 5.38 ±0.18 | 5.38 ± 0.14 | 5.41 ± 0.14 | 5.27 ± 0.11 |

**SUPPLEMTENTARY TABLE S3**

**Supplementary Table S3. Comparison of urine electrolytes excretion and food consumption before the experiment (baseline) and during the experiment days (3 i.p. injection of BRL37344 1 mg/kg or vehicle; protocol 3) for CTR and BRL groups.** Data were presented as mean ± SEM using paired *t* test for each group. **P<0.01, ****P<0.0001.The analysis showed that urine chemistry and food consumption were very similar before the treatment in both groups, and it did not changed by i.p. injection of vehicle.

|  |  | **CTR** | | **BRL** | |
| --- | --- | --- | --- | --- | --- |
|  |  | **BASELINE** | **6 i.p. Vehicle (protocol 4)** | **BASELINE** | **6 i.p. BRL 1 mg/kg (protocol 4)** |
| **I urine collection after I i.p. (CTR=5; BRL =10)** | **uNa+/ucr (mEq/mg)** | 0.32 ± 0.02 | 0.33 ± 0.05 | 0.34 ± 0.04 | 0.17 ± 0.04 ** |
|  | **uK+/ucr (mEq/mg)** | 0.72 ± 0.06 | 0.74 ± 0.06 | 0.75 ± 0.03 | 0.38 ± 0.04 **** |
|  | **uCl-/ucr (mEq/mg)** | 0.63 ± 0.04 | 0.60 ± 0.04 | 0.61 ± 0.06 | 0.24 ± 0.04 **** |
|  | **ucr mg/dl** | 2.49 ± 0.63 | 2.27 ± 0.63 | 2.35 ± 0.51 | 6.56 ± 1.13 ** |
|  | **ucr/urine output (mg)** | 0.08 ± 0.01 | 0.085 ± 0.01 | 0.08 ± 0.04 | 0.07 ± 0.01 |
| **II urine collection after II i.p. (CTR=5; BRL =11)** | **uNa+/ucr (mEq/mg)** | 0.39 ± 0.04 | 0.38 ± 0.09 | 0.36 ± 0.05 | 0.19 ± 0.02 ** |
|  | **uK+/ucr (mEq/mg)** | 0.82 ± 0.05 | 0.78 ± 0.11 | 0.77 ± 0.04 | 0.53 ± 0.06 ** |
|  | **uCl-/ucr (mEq/mg)** | 0.75 ± 0.03 | 0.72 ± 0.14 | 0.74 ± 0.1 | 0.37 ± 0.04 ** |
|  | **ucr mg/dl** | 2.28 ± 1.30 | 2.43 ± 0.70 | 2.5 ± 0.60 | 5.02 ± 1.21 |
|  | **ucr/urine output (mg)** | 0.14 ± 0.02 | 0.13 ± 0.004 | 0.11 ± 0.01 | 0.12 ± 0.01 |
| **III urine collection after III i.p. (CTR=5; BRL =11)** | **uNa+/ucr (mEq/mg)** | 0.82 ± 0.04 | 0.75 ± 0.05 | 0.71 ± 0.06 | 0.52 ± 0.05 * |
|  | **uK+/ucr (mEq/mg)** | 1.28 ± 0.03 | 1.30 ± 0.05 | 1.24 ± 0.03 | 0.98 ± 0.05 *** |
|  | **uCl-/ucr (mEq/mg)** | 1.37 ± 0.09 | 1.40 ± 0.13 | 1.42 ± 0.11 | 1.05 ± 0.07 * |
|  | **ucr mg/dl** | 1.16 ± 0.29 | 1.18 ± 0.30 | 1.35 ± 0.60 | 2.29 ± 0.41 |
|  | **ucr/urine output (mg)** | 0.14 ± 0.02 | 0.12 ± 0.01 | 0.11 ± 0.03 | 0.13 ± 0.01 |
| **IV urine collection after IV i.p. (CTR=5; BRL =11)** | **uNa+/ucr (mEq/mg)** | 0.96 ± 0.14 | 0.94 ± 0.15 | 0.95 ± 0.25 | 0.97 ± 0.06 |
|  | **uK+/ucr (mEq/mg)** | 0.98 ± 0.06 | 0.96 ± 0.05 | 0.96 ± 0.05 | 0.92 ± 0.03 |
|  | **uCl-/ucr (mEq/mg)** | 1.24 ± 0.09 | 1.28 ± 0.23 | 1.24 ± 0.33 | 1.23 ± 0.08 |
|  | **ucr mg/dl** | 1.32 ± 0.29 | 1.35 ± 0.38 | 1.65 ± 0.28 | 1.88 ± 0.22 |
|  | **ucr/urine output (mg)** | 0.17 ± 0.02 | 0.17 ± 0.02 | 0.16 ± 0.04 | 0.18 ± 0.01 |
| **V urine collection after V i.p. (CTR=5; BRL =11)** | **uNa+/ucr (mEq/mg)** | 0.69 ± 0.03 | 0.73 ± 0.04 | 0.75 ± 0.03 | 0.58 ± 0.06 * |
|  | **uK+/ucr (mEq/mg)** | 0.92 ± 0.09 | 0.90 ± 0.09 | 0.81 ± 0.06 | 0.49 ± 0.03 *** |
|  | **uCl-/ucr (mEq/mg)** | 1.09 ± 0.09 | 1.02 ± 0.11 | 1.02 ± 0.09 | 0.75 ± 0.07 * |
|  | **ucr mg/dl** | 1.84 ± 0.98 | 1.63 ± 0.43 | 1.56 ± 0.5 | 3.33 ± 0.64 * |
|  | **ucr/urine output (mg)** | 0.07 ± 0.02 | 0.08 ± 0.02 | 0.07 ± 0.04 | 0.06 ± 0.01 |
| **VI urine collection after VI i.p. (CTR=5; BRL =11)** | **uNa+/ucr (mEq/mg)** | 0.70 ± 0.09 | 0.72 ± 0.31 | 0.77 ± 0.03 | 0.43 ± 0.04 **** |
|  | **uK+/ucr (mEq/mg)** | 0.62 ± 0.07 | 0.56 ± 0.10 | 0.66 ± 0.07 | 0.37 ± 0.03 ** |
|  | **uCl-/ucr (mEq/mg)** | 0.94 ± 0.08 | 0.86 ± 0.30 | 0.96 ± 0.1 | 0.61 ± 0.05 ** |
|  | **ucr mg/dl** | 2.53 ± 1.18 | 2.69 ± 0.90 | 2.56 ± 0.52 | 4.67 ± 0.66 * |
|  | **ucr/urine output (mg)** | 0.03 ± 0.01 | 0.04 ± 0.002 | 0.03 ± 0.005 | 0.04 ± 0.004 |
| **24h (CTR=5; BRL =11)** | **uNa+/ucr (mEq/mg)** | 0.82 ± 0.07 | 0.77 ± 0.13 | 0.87 ± 0.08 | 0.57 ± 0.03 ** |
|  | **uK+/ucr (mEq/mg)** | 1.11 ± 0.06 | 1.10 ± 0.16 | 1.15 ± 0.11 | 0.76 ± 0.04 ** |
|  | **uCl-/ucr (mEq/mg)** | 1.24 ± 0.08 | 1.34 ± 0.27 | 1.44 ± 0.14 | 0.90 ± 0.06 ** |
|  | **ucr mg/dl** | 1.39 ± 0.63 | 1.45 ± 0.48 | 1.51 ± 0.33 | 2.60 ± 0.35 * |
|  | **ucr/urine output (mg)** | 0.56 ± 0.09 | 0.55 ± 0.07 | 0.56 ± 0.05 | 0.59 ± 0.03 |
|  | **food intake (g)** | 5.21 ± 0.29 | 5.10 ± 0.30 | 5.00 ± 0.29 | 4.86 ± 0.14 |

**SUPPLEMENTARY TABLE S4**

**Supplementary Table S4. Comparison of urine electrolytes excretion and food consumption before the experiment (baseline) and during the experiment day (6 i.p. injection of BRL37344 1 mg/kg or vehicle; protocol 4) for CTR and BRL groups.** Data were presented as mean ± SEM using paired *t* test for each group. *P<0.05, **P<0.01, ***P<0.001, ****P<0.0001.

Data indicated that only and exclusively BRL treatment modified the urine concentration of sodium, potassium, chloride.

**References**

1. Susulic VS, Frederich RC, Lawitts J, et al. Targeted disruption of the beta 3-adrenergic receptor gene. *The Journal of biological chemistry*. Dec 8 1995;270(49):29483-92. <https://doi.org10.1074/jbc.270.49.29483>.
